# In Situ Elucidations of the Mechanism for Inert Molecular N_2_O Decomposition at Low Temperature Catalyzed by High‐Index Interfacial Exposed Co_3_O_4_

**DOI:** 10.1002/advs.74735

**Published:** 2026-03-09

**Authors:** Lei Pan, Qian Mi, Minghao Wang, Luyu Wang, Yiyang Xie, Huanjie Zhang, Ruinian Xu, Biaohua Chen

**Affiliations:** ^1^ College of Environmental Science and Engineering Beijing University of Technology Beijing China; ^2^ Material Science and Engineering Beijing University of Technology Beijing China; ^3^ Key Laboratory of Optoelectronics Technology Ministry of Education Beijing University of Technology Beijing China

**Keywords:** Co_3_O_4_, high‐index facets, interfacial facet, mechanisms, N_2_O decomposition

## Abstract

Co_3_O_4_ has emerged as a promising catalyst for decomposition of greenhouse gas N_2_O (*de*N_2_O). And the catalytic performance can be significantly influenced by the different exposed crystalline facets, with multiple high‐index facets garnering attention because of their superior activity. However, challenges persist in the synthesis of high‐index facets and in fully understanding their catalytic mechanisms. This work introduces a straightforward method for synthesizing multi high‐index crystal facet catalysts and establishes the mechanism of low‐temperature *de*N_2_O catalysis through in situ experiments and theoretical calculations. Compared to commonly synthesized Co_3_O_4_ with the (110) facet exposed, the as‐synthesized Co_3_O_4_ with the interfacial facet (400−400), performs nearly 8 times higher on *de*N_2_O efficiency at 300°C. Experimental results and density functional theory calculations demonstrate that Co^3+^ on (400−400) can form a novel active Co^3+^‐O^*^ motif during the *de*N_2_O reaction, which exhibits a unique electronic structure and offers an alternative route for *de*N_2_O, effectively shortening the reaction steps and enhancing overall efficiency. This work establishes an essential theoretical foundation for the development of catalysts capable of activating inert molecules at low temperatures, thereby enabling their activation and utilization.

## Graphical abstract

This study develops a co‐precipitation followed by hydrothermal synthesis strategy for synthesizing Co_3_O_4_ catalyst with multiple high‐index facets. Experiments and density functional theory calculations demonstrate that Co^3+^ on the (400–400) facet can form a novel active Co^3+^‐O^*^ motif during the reaction. This motif features a distinct electronic structure and enables a shorter, more efficient reaction pathway.

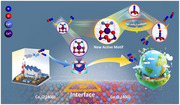

## 1 Introduction

**FIGURE 1:**
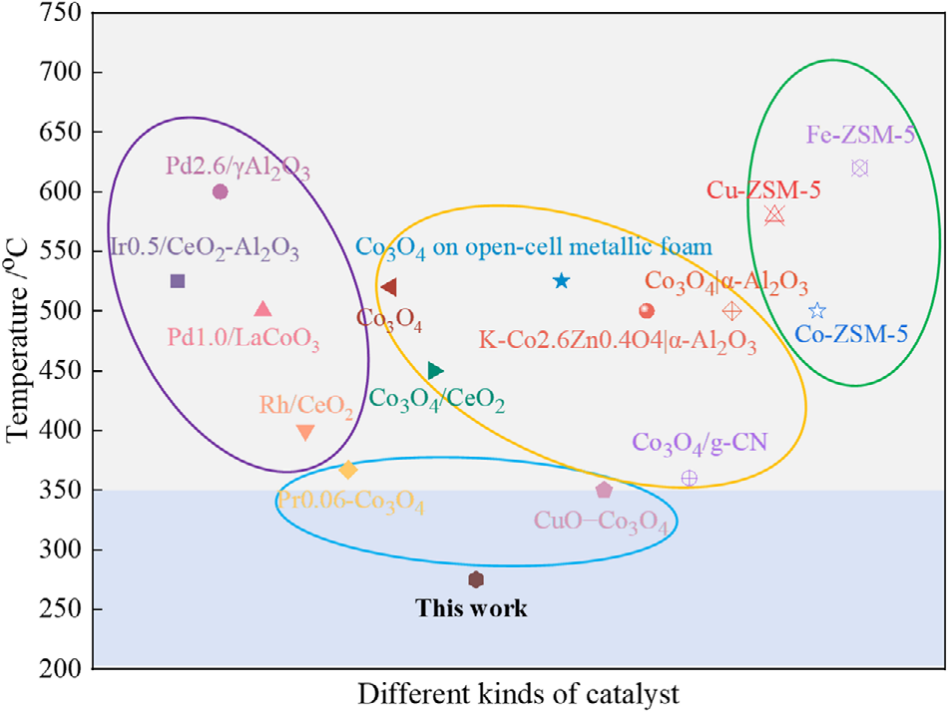
*T*_90_ values for *de*N_2_O conversion on various catalysts sourced from previous literatures (as listed in Table S1).

Nitrous Oxide (N_2_O), the third most significant greenhouse gas, possesses a warming potential approximately 298 times greater than that of CO_2_ and is expected to remain the primary ozone‐depleting substance throughout the 21st century, causing harm to human beings [1, 2, 3]. Currently, direct catalytic decomposition of N_2_O to N_2_ and O_2_ is one of the most promising post‐treatment technologies [4, 5, 6, 7]. Catalysts are essential to this process, and numerous catalysts have been developed for this purpose. Despite variations in catalysts and experimental conditions, it was found that *T*_90_ value is generally above 350°C (Figure 1; Table S1). The primary reason for this phenomenon is the inert nature of the N_2_O molecule: the stable N≡N and N═O bonds in N_2_O result in a high bond dissociation energy, posing significant challenges for catalysts to achieve activation and bond cleavage at low temperatures. Among various catalysts, Co_3_O_4_ exhibits relatively superior low‐temperature activity and has thus attracted significant attention as a promising catalytic material.

To effectively address this challenge, significant efforts were directed toward developing catalysts with high electron‐transfer capabilities, which are essential for reducing the bond breaking energy of N_2_O. Most recently, various strategies, including structural manipulation [8], defect engineering [9], and facet engineering [10], have been proposed on metallic catalysts to enhance the electron transfer efficiency of catalysts. It finds that adjusting the crystal facets of metallic catalysts can significantly enhance the activation of those molecules with strong chemical bonds such as NO/N_2_O (N═O) [11, 12]. CH_4_ (C─H) [13], and CO_2_ (C═O) [14]. The facet structure governs the geometric and electronic properties of active sites, modulates the adsorption configurations and energy barriers of reaction intermediates, and serves as a fundamental intrinsic parameter for tailoring catalytic reaction pathways and efficiency [15]. Compared with low‐index crystal facets, high‐index crystal facets possess higher surface energy and stronger coordination unsaturation, significantly regulating the electronic state density and surface reactivity of the material, thereby endowing it with superior catalytic functional potential [16]. In reactions such as the low‐temperature decomposition of N_2_O, it has been demonstrated that exposing high‐index crystal facets can significantly reduce the activation energy of the reaction. For example, Xiong et al. [17] prepared Gd‐promoted Co_3_O_4_ catalysts, promoting exposure to a greater variety of high‐index facets such as (400), (440), and (511). This modification resulted in a reduction of the *T*_90_ from 450°C to 350°C in a 0.2 vol.% N_2_O/Ar.

However, due to the high surface free energy of high‐index crystal facets, they are thermodynamically unstable, and their selective exposure faces severe challenges. The existing synthesis methods mostly rely on adding organic solvents, metal doping, heterostructure construction, and chemical etching [18, 19, 20], which not only involve cumbersome processes and high costs but also are difficult to achieve large‐scale production, seriously restricting their practical application and mechanism exploration. Utilizing Monte Carlo simulations, Akutsu et al. [21] investigated the processes of nucleation and crystal growth. Their findings indicate that elevated temperatures disrupt thermodynamic equilibrium, thereby facilitating crystal growth. Bugaris et al. [22] developed a method for growing crystals from high‐temperature solutions, which they assert is optimal for discovering novel, complex structured components with potentially new properties. Consequently, the hydrothermal method has shown potential in exposing high‐index crystal facets due to its excellent ability to control the crystal growth direction. Guo et al. [23] successfully synthesized platinum nanoparticles with high‐index crystal facets, specifically (310), (520), and (830), by employing a one‐pot hydrothermal method. Huang et al. [24] also successfully synthesized materials with exposed high‐index crystal facets (400) of Fe_3_O_4_ through a hydrothermal method, and found that the oriented interface could effectively promote the oxygen evolution reaction and significantly reduce the free energy barrier of the rate‐limiting step of the reaction. However, for Co‐based oxides, especially the controllable construction of those with high‐index crystal facets, there is still a lack of simple, efficient and scalable synthetic strategies. More notably, the interface itself, as a region of electronic structure reconstruction and localized strain concentration, often forms highly active sites [25]. Zhang et al. [26] significantly enhanced catalytic performance by constructing an internal electric field to stabilize the high‐valent Co^3+^ active centers in Co_3_O_4_, revealing the crucial role of interface engineering in activity regulation. However, most current studies focus on crystal facet exposure while neglecting the active design and synergetic effects of grain boundary interfaces, especially in the regulation of interfaces between high‐index crystal facets, which remains an unexplored area.

In response to the above challenges, we propose a simple and scalable two‐step co‐precipitation + hydrothermal synthesis strategy. By stepwise regulation of the nucleation and crystal growth kinetics, we achieve selective exposure of high‐index crystal facets (particularly the (400) facet) in Co_3_O_4_ and successfully induce the formation of (400–400) grain boundary interfaces. This method does not require complex templates or expensive additives. Through systematic optimization of the precursor concentration, pH value, and hydrothermal conditions, we effectively control the crystal morphology and interface structure. The prepared Co_3_O_4_‐C+H catalyst exhibits exceptional performance in *de*N_2_O at low temperature, achieving more than 98.1% conversion at 300°C in a 7 vol.% N_2_O, 2 vol.% H_2_O /He mixture (Figure 1). Furthermore, utilizing comprehensive characterizations and multiple in situ techniques, alongside density functional theory calculations, this work examine the underlying mechanisms responsible for the enhanced *de*N_2_O performance demonstrated by the interfacial facet (400−400) on Co_3_O_4_‐C+H at low temperatures. The results not only address three critical questions concerning the application of Co_3_O_4_‐C+H for the *de*N_2_O reaction: i) What kind of Co_3_O_4_ facet is optimal for *de*N_2_O reaction? ii) What is the key for the low‐temperature effective *de*N_2_O? iii) What factor determines the adsorption manner and subsequent decomposition pathway of N_2_O molecules? This work not only provides a new approach for the controllable synthesis of high surface energy crystal facets, but more importantly, establishes a direct connection between “synthesis strategy‐crystal facet/interface construction‐catalytic performance”, highlighting the core position of interfaces in the design of catalytic active sites, and offers a practical example for the rational design of high‐performance transition metal oxide catalysts.

## 2 Results and Discussion

### 2.1 *De*N_2_O Performance Over Co_3_O_4_ Catalysts

**FIGURE 2:**
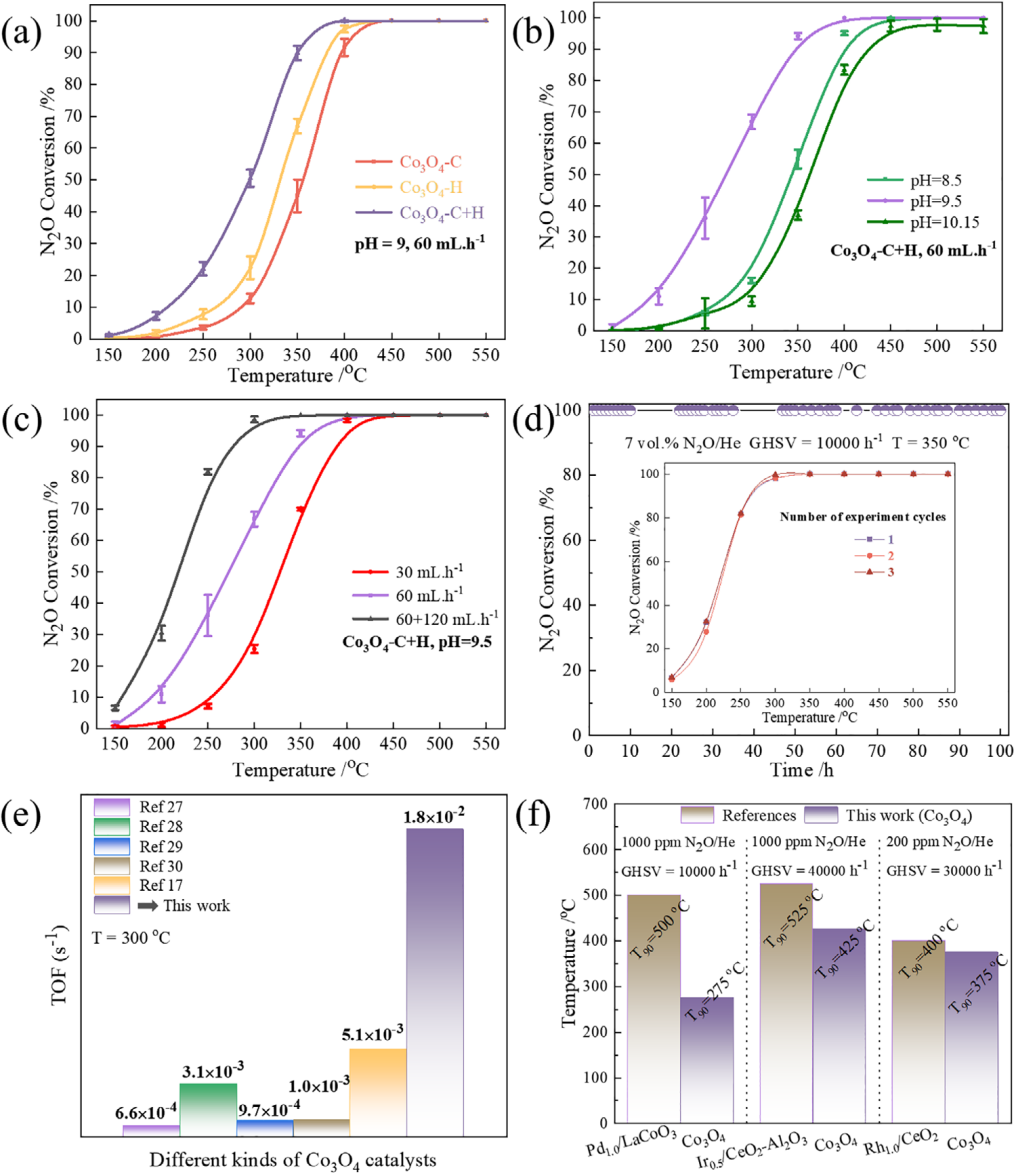
Performance evaluation and comparison of a series of Co_3_O_4_ catalysts under reaction conditions of 7 vol.% N_2_O, 2 vol.% H_2_O, with He as the balance gas and GHSV of 10 000 h^−1^. (a) The N_2_O conversion of Co_3_O_4_‐C, Co_3_O_4_‐H, and Co_3_O_4_‐C+H catalysts. (b) The N_2_O conversion of Co_3_O_4_‐C+H catalysts prepared at different pH values under a Na_2_CO_3_ flow rate of 60 mL/h. (c) The N_2_O conversion of Co_3_O_4_‐C+H catalysts prepared under different flow rates of Na_2_CO_3_ at pH 9.5. (d) Reliability and long‐term stability test of the best Co_3_O_4_‐C+H catalyst. (e) The TOF comparison of Co_3_O_4_ catalysts prepared by different methods at 300°C. (f) The T_90_ of Co_3_O_4_ and noble metal catalysts under the same working conditions.

This work systematically investigated the effects of different preparation methods on the low‐temperature catalytic decomposition performance of N_2_O over Co_3_O_4_ catalysts, including the co‐precipitation method (Co_3_O_4_‐C) and the one‐step hydrothermal method (Co_3_O_4_‐H), and evaluated their activities under reaction conditions of 7 vol.% N_2_O, 2 vol.% H_2_O, He as the balance gas, and GHSV = 10 000 h^−1^. As shown in Figure 2a, the conversion rate of Co_3_O_4_‐C was only about 45% at 350°C, while that of Co_3_O_4_‐H increased to about 67%, and Co_3_O_4_‐C+H reached 90%, significantly outperforming the single methods, fully demonstrating the advantages of the two‐step method in structure regulation and performance optimization. All Co_3_O_4_ catalysts displayed N_2_ selectivity as high as 99.99% during the catalytic tests (Figure S1).

Further, we examined the effects of the pH value and the drop rate of the precipitating agent on the performance of Co_3_O_4_‐C+H (Figure S2). The catalytic activity increased first and then decreased with the increase of pH, reaching the optimum at pH = 9.5, with a N_2_O conversion rate of 94% at 350°C (Figure 2b). The drop rate also showed a similar trend, with the sample prepared at a mixed drop rate (60 + 120 mL/h) having the best performance, achieving 100% conversion at 350°C (Figure 2c). Long‐term stability tests (100 h) and cycling experiments indicated that Co_3_O_4_‐C+H maintained high activity and structural stability at low temperatures (Figure 2d). Additionally, this catalyst demonstrated excellent tolerance in high oxygen concentration (10 vol.% O_2_) and high humidity atmosphere (10 vol.% H_2_O), maintaining high conversion efficiency at 350°C, showing great potential for industrial application (Figure S3). Compared with various Co_3_O_4_ catalysts reported in the literature (Figure 2e) and noble metal systems (Figure 2f) [27, 28, 29, 30, 31, 32, 33], the Co_3_O_4_‐C+H in this work not only has a higher TOF value but also shows outstanding performance in low‐temperature activity, stability, and interference resistance, highlighting its significant advantages as a highly efficient non‐noble metal N_2_O decomposition catalyst.

### 2.2 Structural Characterization of Co_3_O_4_ Catalysts

**FIGURE 3:**
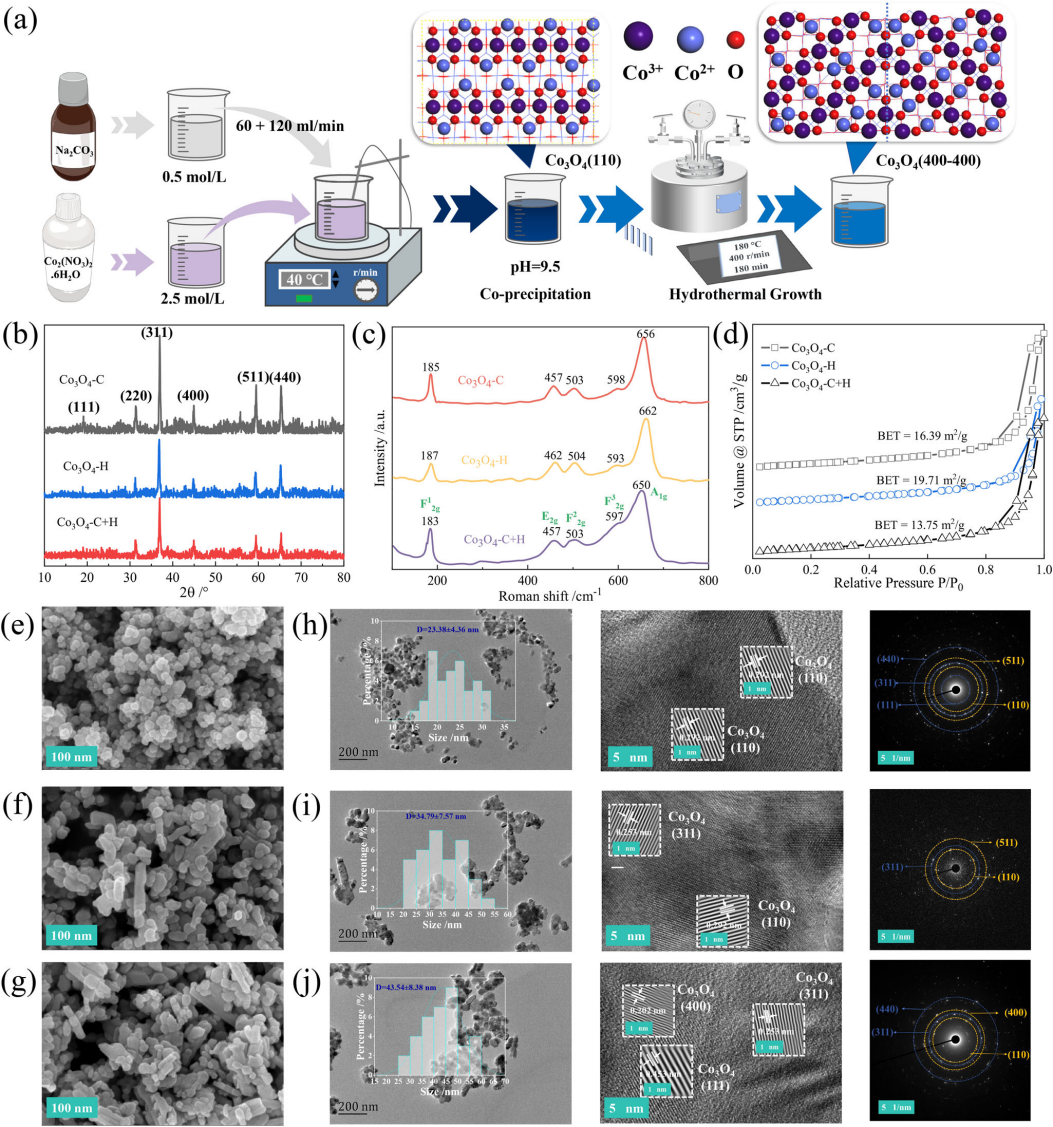
The synthesis process and basic characterization of Co_3_O_4_ catalysts. (a) Synthesis process of Co_3_O_4_‐C+H catalysts. (b) XRD patterns of Co_3_O_4_‐C, Co_3_O_4_‐H, and Co_3_O_4_‐C+H catalysts. (c) Roman spectra of Co_3_O_4_‐C, Co_3_O_4_‐H, and Co_3_O_4_‐C+H catalysts. (d) N_2_ adsorption‐desorption isotherms of Co_3_O_4_‐C, Co_3_O_4_‐H, and Co_3_O_4_‐C+H catalysts. (e–g) SEM images of Co_3_O_4_‐C, Co_3_O_4_‐H, and Co_3_O_4_‐C+H catalysts. (h–j) TEM‐SAED images of Co_3_O_4_‐C, Co_3_O_4_‐H, and Co_3_O_4_‐C+H catalysts.

Figure 3a shows a schematic diagram of the two‐step preparation method, which includes two key steps: co‐precipitation and hydrothermal treatment, corresponding to the formation of crystal nuclei and the growth of crystal facets, respectively. Therefore, the excellent catalytic performance of the Co_3_O_4_‐C+H catalyst is closely related to its unique structural features and crystal facet regulation. This subsection focuses on Co_3_O_4_ catalyst samples prepared by different methods and selects Co_3_O_4_‐C+H samples prepared at different drop rates to systematically reveal the influence of synthesis conditions on the microstructure and crystal facet exposure behavior of the catalyst. As illustrated in X‐ray diffraction (XRD) patterns, the six characteristic peaks correspond perfectly to Co_3_O_4_ (PDF# 43–1003) phases. (Figure 3b) [34]. However, the noticeably weaker crystallinity of Co_3_O_4_‐C+H suggests a reconstruction of its crystal surface structure. Raman spectrum can give out the defect structure properties of catalysts (Figure 3c), including tetrahedral sites Co^2+^‐O^2−^ (F^1^_2g_, E_2g_) and octahedral sites Co^3+^‐O^2−^ (F^2^_2g_, F^3^_2g_, A_1g_). The peak of the A_1g_ vibration mode of Co_3_O_4_‐C+H exhibited a red shift from 656 and 662 to 650 cm^−1^, indicating that the two‐step treatment significantly affects catalyst morphology, promoting more crystal defects [35]. The porous structure and specific surface area of the synthesized composites were further analyzed by the N_2_ adsorption‐desorption isotherms. Figure 3d presented typical type IV isotherm with a hysteresis loop type H3, proving the mesoporous textures exist in all Co_3_O_4_ catalysts(Table S2).

Moreover, scanning electron microscopy (SEM) images (Figure 3e–g) revealed clear differences in micro‐morphology among Co_3_O_4_‐C, Co_3_O_4_‐H, and Co_3_O_4_‐C+H, with hydrothermally synthesized catalysts exhibiting larger particle sizes. And transmission electron microscopy (TEM) images showed that the particle size of Co_3_O_4_‐C+H was ∼44 nm larger than that of Co_3_O_4_‐H as ∼35nm and Co_3_O_4_‐C as ∼24nm (Figure 3h–j). Note that the lattice spacings of 0.292 nm are related to (110) facet of Co_3_O_4_; while that of 0.202, 0.253, and 0.453 nm are corresponded to (400), (311), and (111) facets of Co_3_O_4_, respectively [36, 37, 38]. The TEM‐SAED analysis indicates that Co_3_O_4_‐C presents (111), (110), (311), (511), and (440) crystal facets, consistent with the XRD results; Co_3_O_4_‐H mainly exposes (110), (311), and (511) crystal facets; while the two‐step prepared Co_3_O_4_‐C+H shows (110), (311), (400), and (440) crystal facets, with a higher proportion and more concentrated distribution of high‐index crystal facets, suggesting that the synthesis method significantly affects the crystal facet exposure orientation, and the two‐step method is conducive to the directional formation of specific high‐activity crystal facets.

**FIGURE 4:**
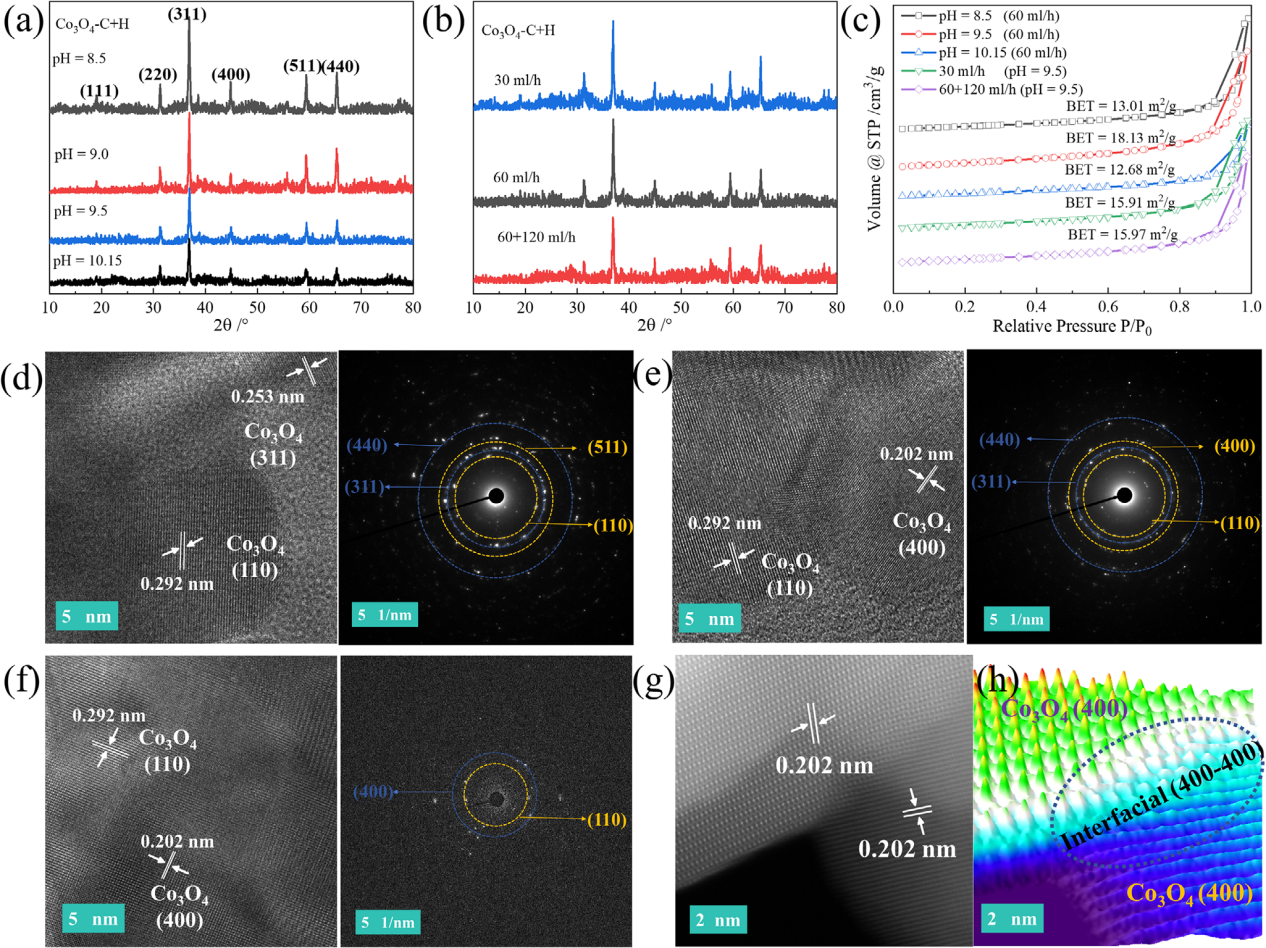
The characterization of Co_3_O_4_‐C+H catalysts prepared by different control methods. (a) XRD patterns of Co_3_O_4_‐C+H catalysts prepared at different pH values under a Na_2_CO_3_ flow rate of 60 mL/h. (b) XRD patterns of Co_3_O_4_‐C+H catalysts prepared under different flow rates of Na_2_CO_3_ at pH 9.5. (c) N_2_ adsorption‐desorption isotherms of Co_3_O_4_‐C+H catalysts. (d–f) TEM‐SAED images of Co_3_O_4_‐C+H (30 mL/h, pH = 9.5); Co_3_O_4_‐C+H (60 mL/h, pH = 9.5); Co_3_O_4_‐C+H (60+120 mL/h, pH = 9.5). (g) HAADF‐STEM image of the best Co_3_O_4_‐C+H (60+120 mL/h, pH = 9.5) catalyst. (h) 3D rendering of HAADF‐STEM image.

Based on the two‐step method, the optimal conditions including types and amounts of precipitant and drop acceleration were thoroughly determined based on performance evaluation results (Figure S2). Notably, precipitating agent dosage and addition rate significantly influenced material performance. The XRD patterns confirm that all catalysts exhibit the six characteristic peaks of the Co_3_O_4_ crystal phase (Figure 4a,b), though peak intensity variations indicate changes in crystal structure. All catalysts possess mesoporous structures, with differences in microstructure further evidenced by BET surface area (Figure 4c), and SEM images (Figure S4), particularly in crystal facet features. Further comparison of samples prepared at different drop rates reveals that the sample prepared at 30 mL/h contains (110), (311), (511), and (440) crystal facets; the sample prepared at 60 mL/h presents (110), (311), (400), and (440) crystal facets; while the best sample prepared at a mixed drop rate of 60+120 mL/h only shows (110) and (400) crystal facets (Figure 4d–f). Combined with the catalytic performance data, the repeated appearance of (110), (311), and (400) crystal facets in these samples suggests that they may be closely related to the excellent activity. Additionally, high‐angle annular dark‐field scanning transmission electron microscopy (HAADF‐STEM) analysis was conducted on the optimal sample Co_3_O_4_‐C+H (60+120 mL/h), and the existence of (400–400) grain boundary structures was clearly observed, indicating that this interface may be one of the key active features (Figure 4g,h). These results solidly prove that the two‐step synthesis strategy successfully builds multi‐facets over the Co_3_O_4_‐C+H. According to previous reports [39], the high index facets, especially the interfacial ones, can provide benefits for electron transfer, O^*^ transfer, and reactants activation for reaction of N_2_O decompositio

### 2.3 Character of Multi High‐Index Facets Co_3_O_4_

**FIGURE 5:**
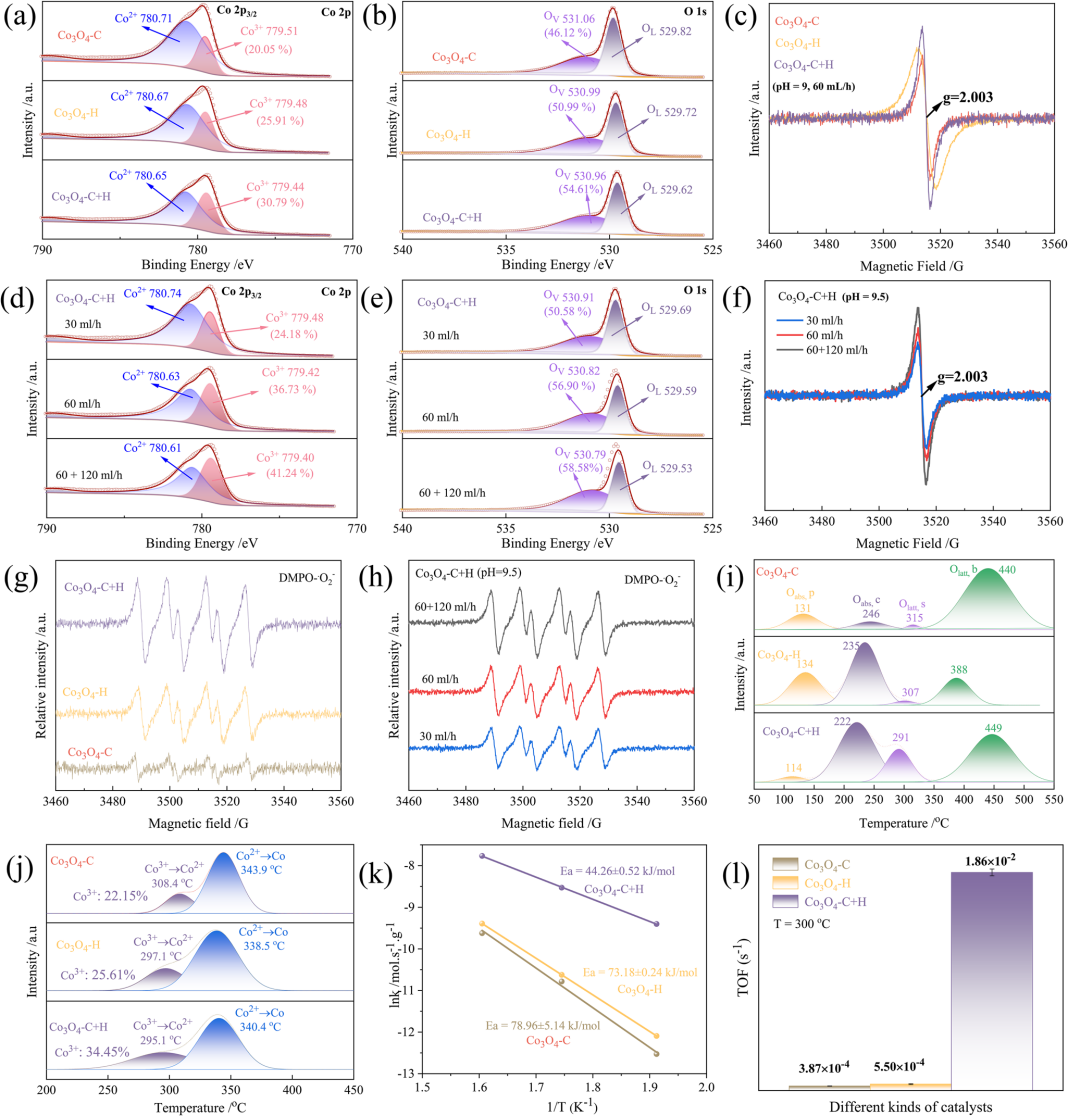
Characterization of active sites of a series of Co_3_O_4_ catalysts. (a–c) The XPS spectra of Co 2p, O 1s, and EPR of Co_3_O_4_ catalysts prepared by different methods under the conditions of pH = 9 and a flow rate of 60 mL/h. (d–f) The XPS spectra of Co 2p, O 1s, and EPR of Co_3_O_4_‐C+H catalysts prepared by different dripping speeds under the conditions of pH = 9.5. (g) EPR tests on ^.^O_2_^−^ of Co_3_O_4_ catalysts prepared by different methods under the conditions of pH = 9 and a flow rate of 60 mL/h. (h) EPR tests on ^.^O_2_^−^ of Co_3_O_4_‐C+H catalysts prepared by different dripping speeds under the conditions of pH = 9.5. (i,j) The O_2_‐TPD and H_2_‐TPR spectra of Co_3_O_4_‐C, Co_3_O_4_‐H and the optimal Co_3_O_4_‐C+H catalysts. (k,l) Ea and TOF of Co_3_O_4_‐C, Co_3_O_4_‐H and the optimal Co_3_O_4_‐C+H catalysts.

To elucidate the evaluation of surface valence states and the electronic structures of Co_3_O_4_ samples, the X‐ray photoelectron spectroscopy (XPS) measurement was conducted. The Co 2p_3/2_ orbitals are displayed in Figure 5a, with two peaks at binding energies of 779.5 and 780.7 eV belonging to Co^3+^ and Co^2+^, respectively [40]. The results show that, compared with Co_3_O_4_‐C prepared by coprecipitation, the Co 2p_3/2_ main peak of Co_3_O_4_‐H and Co_3_O_4_‐C+H obtained by hydrothermal treatment shifts significantly toward lower binding energy. This phenomenon indicates that the hydrothermal process induces an increase in surface electron density, possibly due to lattice distortion, oxygen vacancy formation or weakening of the Co─O bond, thereby reducing the binding energy of core electrons. Quantitative analysis further reveals that the relative content of Co^3+^ is the highest in Co_3_O_4_‐C+H at 30.79% compared to 25.91% in Co_3_O_4_‐H and 20.05% in Co_3_O_4_‐C, indicating that the two‐step method not only regulates the exposure of high‐index crystal facets but also optimizes the valence state distribution of active metal centers, which is conducive to enhancing the redox ability. The O 1s spectra of different samples in Figure 5b demonstrated the presence of two distinct oxygen species on the catalyst surface. The peak at 529.8 and 531.1 eV in Co_3_O_4_ can be attributed to the lattice oxygen (O_L_) and surface‐adsorbed oxygen species on oxygen vacancies (O_V_), respectively. The lattice oxygen peak also shows a similar shift toward lower binding energy, indicating that the hydrothermal treatment alters the surface chemical environment and enhances the mobility and reactivity of lattice oxygen [41]. The relative content of O_v_ is the highest in Co_3_O_4_‐C+H than Co_3_O_4_‐H and Co_3_O_4_‐C, which is highly consistent with the electron paramagnetic resonance (EPR) results (Figure 5c), confirming that the two‐step method effectively promotes the construction of surface defects.

In addition, the binding energy of Co 2p_3/2_ in Co_3_O_4_‐C+H is significantly affected by different pH values and drop rates (Figure S5d). XPS analysis shows that the binding energy of Co 2p_3/2_ is the lowest in the sample prepared at pH = 9.5 and with the optimal drop rate (60 + 120 mL/h) at this pH, indicating a higher electron density of surface Co^3+^ and enhanced metal‐oxygen covalency, which is conducive to promoting charge transfer and thus enhancing catalytic activity. Meanwhile, the Co^3+^ content is highest at pH 9.5, reaching 36.73%, and under the optimal drop rate, it increases to 41.24%, the highest among all conditions, suggesting that high‐index crystal facets may expose more Co^3+^ sites, thereby enhancing surface reactivity. This trend is consistent with the catalytic performance results, suggesting that regulating the synthesis conditions can optimize the catalyst's electronic structure and thereby enhance its intrinsic activity. By optimizing the precipitating agent's amount and addition rate, the best‐performing Co_3_O_4_‐C+H achieves the highest oxygen vacancy concentration (∼59%) (Figure 5e). The lower binding energy of the O_L_ component suggests weakened Co─O interactions, which is attributed to the enriched electron density of O_L_ caused by the existence of oxygen vacancies. EPR spectra (Figure 5f) revealed additional verification of abundant oxygen vacancies (represented as a pair of stronger peaks with a symmetric distribution at *g* = 2.003) for the best‐performing Co_3_O_4_‐C+H. Superoxide radicals (^.^O_2_^−^) act as key intermediates in the migration and desorption of O^*^ species, which can accelerate this process. EPR characterization using 5,5‐dimethyl‐1‐pyrroline N‐oxide (DMPO) as the spin trap was performed to detect and compare superoxide radical generation over six catalysts prepared via different methods (Figure 5g) and varying drop rates (Figure 5h). As illustrated in Figure 5g, the signal intensity of superoxide radicals (^.^O_2_^−^) varied notably among catalysts synthesized by different preparation methods: the Co_3_O_4_‐C sample displayed the weakest ^.^O_2_^−^ signal, the Co_3_O_4_‐H showed a moderate signal, and the Co_3_O_4_‐C+H catalyst displayed the strongest ^.^O_2_^−^ signal. Furthermore, the optimal Co_3_O_4_‐C+H sample, prepared using a hybrid dropping rate, also exhibited higher ^.^O_2_^−^ signal intensity (Figure 5h). In conjunction with the catalytic performance test results (Figure 2), it is concluded that a stronger ^.^O_2_^−^ signal facilitates the migration and desorption of surface‐adsorbed oxygen species (O^*^) during N_2_O decomposition, thereby accelerating O_2_ generation and enhancing low‐temperature catalytic activity.

To highlight the advantages of the two‐step process, the following section compares the optimal hydrothermal catalyst (Co_3_O_4_‐C+H) with the classic co‐precipitation method (Co_3_O_4_‐C+H) and the one‐step hydrothermal method (Co_3_O_4_‐H). Oxygen temperature‐programmed desorption (O_2_‐TPD) was carried out to distinguish the oxygen species, and four types of peaks were identified (Figure 5i), including physical adsorption of oxygen (O_abs_,p), chemical adsorbed oxygen (O_abs_,c), surface lattice oxygen (O_latt_,s), and bulk lattice oxygen (O_latt_,b) [42]. Clearly, compared to Co_3_O_4_‐C and Co_3_O_4_‐H, the Co_3_O_4_‐C+H performed remarkably greater O_abs_,c and O_latt_,s desorption signals at a lower temperature. It suggests that the Co─O bond in Co_3_O_4_‐C+H is more prone to cleavage, thereby enhancing the transfer of surface oxygen. Hydrogen temperature‐programmed reduction (H_2_‐TPR) results further confirmed the substantial presence of Co^3+^ in Co_3_O_4_‐C+H (Figure 5j), and the peak reduction temperature of Co^3+^ to Co^2+^ shifted to 295°C (compared to 308°C for Co_3_O_4_‐C and 297°C for Co_3_O_4_‐H), indicating enhanced reducing capacity and a greater number of active Co─O bonds [43]. The relative content of Co^3+^ also reached its peak value, at 34.5%, which was higher than 25.6% of Co_3_O_4_‐H and 22.2% of Co_3_O_4_‐C. The unique features of these high‐index crystal facets enable efficient low‐temperature N_2_O decomposition over Co_3_O_4_‐C+H. Further kinetic analysis indicates that Co_3_O_4_‐C+H has the lowest apparent activation energy of 44.3 kJ/mol, significantly lower than that of Co_3_O_4_‐H (73.2 kJ/mol) and Co_3_O_4_‐C (79.0 kJ/mol) (Figure 5k; Figure S6). At 300°C, its TOF value reaches 1.86 × 10^−2^ s^−1^, which is over 30 times higher than that of Co_3_O_4_‐H (5.50 × 10^−4^ s^−1^) and Co_3_O_4_‐C (3.87 × 10^−4^ s^−1^) (Figure 5l; Table S3), and is superior to other Co_3_O_4_ catalysts prepared by methods reported in the literature (Figure 2e). This result fully confirms the significant advantage of the two‐step method in enhancing intrinsic catalytic activity, which is closely related to the high‐index crystal facets, optimized electronic structure, and favorable surface properties it forms, further verifying the rationality of the structure‐performance relationship.

### 2.4 Monitoring of *de*N_2_O Process Over Co_3_O_4_‐C and Co_3_O_4_‐C+H Catalysts

**FIGURE 6:**
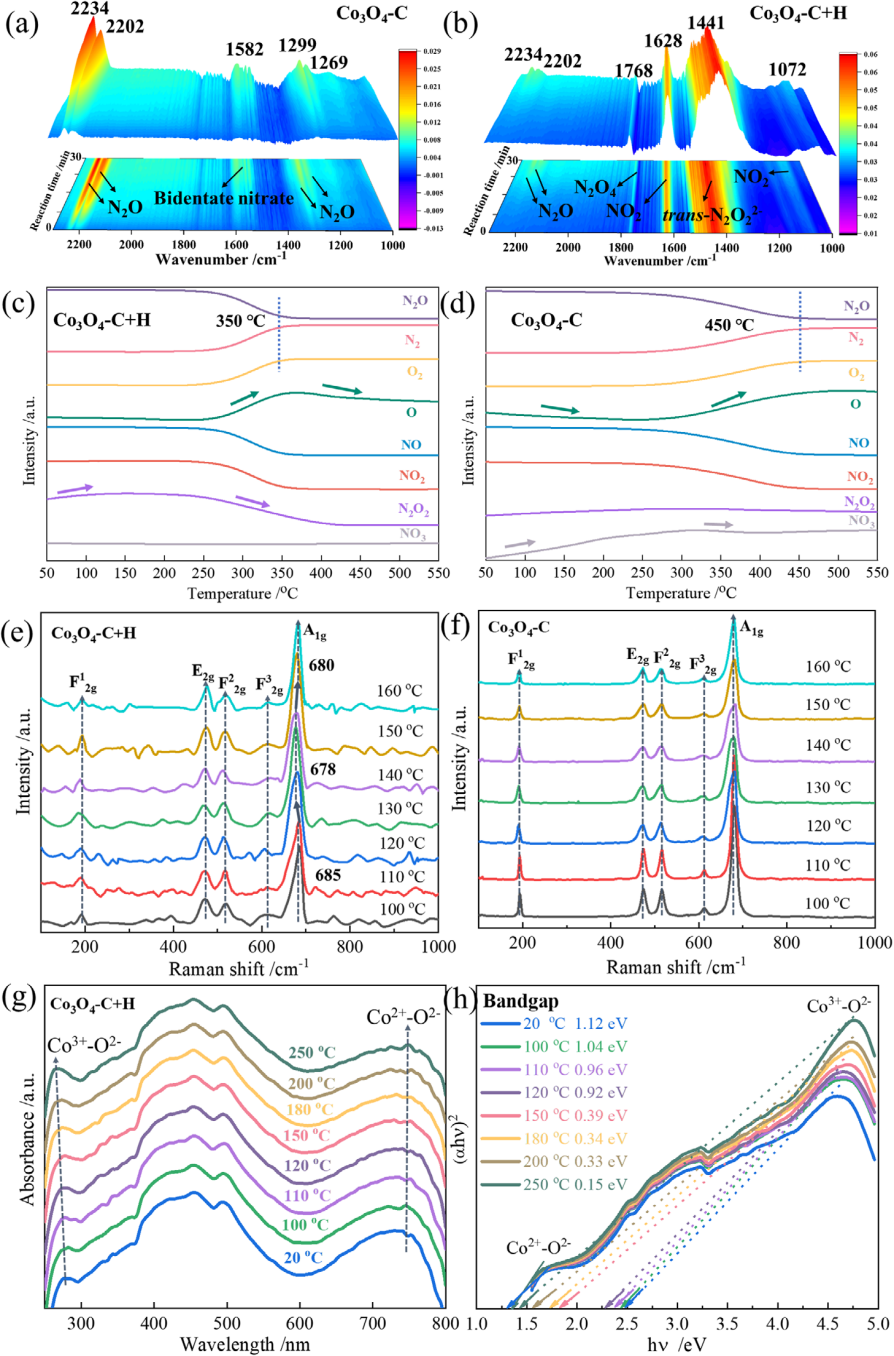
In situ characterization of Co_3_O_4_‐C and Co_3_O_4_‐C+H catalysts for monitoring the decomposition process of N_2_O. (a,b) in situ DRIFTS of Co_3_O_4_‐C and Co_3_O_4_‐C+H catalysts at 250°C in a 7 vol.% N_2_O/He atmosphere. (c,d) The TPSR profiles of Co_3_O_4_‐C+H and Co_3_O_4_‐C catalysts in a 7 vol.% N_2_O/He atmosphere. (e,f) in situ Raman spectra of Co_3_O_4_‐C+H and Co_3_O_4_‐C catalysts in a 7 vol.% N_2_O/He atmosphere. (g,h) in situ UV–vis DRS and bandgaps of Co_3_O_4_‐C+H catalyst in a 7 vol.% N_2_O/He atmosphere.

Based on the aforementioned performance and characterization results, it can be concluded that the catalytic activity and structural characteristics of Co_3_O_4_‐C lie between those of Co_3_O_4_‐H and Co_3_O_4_‐C+H. To more clearly reveal the relationship between structure and performance, this section further focuses on Co_3_O_4_‐C and the optimal sample Co_3_O_4_‐C+H catalysts, systematically studying the adsorption, activation and decomposition of N_2_O on their surfaces through a series of in situ characterization techniques, and deeply analyzing the structural evolution and action mechanism of the catalysts during the reaction process. As shown in Figure 6a, the bands appeared at 2234, 2202, 1299, and 1269 cm^−1^, which are assigned to the stretching vibrations of the N≡N and N─O bonds resulting from N_2_O adsorption at the Co sites on Co_3_O_4_‐C [44]. Additionally, the band at 1582 cm^−1^ is associated with bidentate nitrate. Differently, the characteristic peak intensity of N_2_O in the Co_3_O_4_‐C+H sample is very low, while those of N_2_O_4_ (1768 cm^−1^), NO_2_ (1628, 1072 cm^−1^), bidentate nitrate (1512 cm^−1^), and *trans*‐N_2_O_2_^2−^ (1441 cm^−1^) are obvious (Figure 6b) [45, 46, 47]. This suggests that N_2_O rapidly converts into various intermediates under 250°C, consistent with the high TOF and catalytic performance. The intermediate products show different N_2_O decomposition pathways on Co_3_O_4_‐C and Co_3_O_4_‐C+H catalysts, with the new pathway appearing on Co_3_O_4_‐C+H. In the infrared spectrum of Co_3_O_4_‐H (Figure S7), adsorption peaks of N_2_O and intermediates like NO_2_, nitrate, and *trans*‐N_2_O_2_^2−^ are observed, but their intensities are much lower than on Co_3_O_4_‐C+H. Co_3_O_4_‐H with high‐index crystal facets, and especially Co_3_O_4_‐C+H containing both high‐index facets and grain boundary structures, shows significantly higher catalytic activity for low‐temperature N_2_O conversion than conventional Co_3_O_4_‐C, enabling rapid reaction initiation and intermediate formation. This suggests a strong correlation between enhanced catalytic performance and the unique high‐index crystal facet structure.

Temperature‐programmed surface reaction (TPSR) experiments were used to monitor the reaction process involved in the decomposition of N_2_O on Co_3_O_4_ catalyst surfaces. As depicted in Figure 6c,d, the N_2_O signal decreases as the reaction temperature rises from 150°C to 550°C in a 7 vol.% N_2_O/He atmosphere. This observation aligns with the activity test results. Simultaneously, substantial amounts of N_2_ and O_2_ were produced, indicating the reaction 2N_2_O → O_2_ + 2N_2_ occurred. Furthermore, the O signal curve of Co_3_O_4_‐C+H closely follows the O_2_ signal curve, with deviations occurring only in the high‐temperature region (Figure 6c). This indicates that the formation of O^*^ from the decomposition of N_2_O potentially contributes to O_2_ formation through various reaction pathways, one possibly involving the intermediate *trans*‐N_2_O_2_^2−^. And the *trans*‐N_2_O_2_^2−^ signal exhibited a significant decrease at elevated temperatures, further corroborating the *de*N_2_O process involving *trans*‐N_2_O_2_^2−^ species. In contrast, the O signal curve of Co_3_O_4_‐C fails to align with the O_2_ signal, displaying significant fluctuations at low temperatures (Figure 6d). This suggests that O^*^ may engage in alternative reactions. Notably, the −NO_3_ signal curve shows a pronounced increase at low temperatures, which not only corroborates its presence and concurs with findings from the infrared analysis but also suggests that the production of nitrates is likely derived from O^*^. Apparently, it can deduce that the exceptional reaction behavior and outstanding *de*N_2_O performance for Co_3_O_4_‐C+H are intrinsically associated with its unique high‐index facets.

Therefore, in situ Raman spectra were recorded to further explore the effect of active phases during *de*N_2_O process. Raman spectra for Co_3_O_4_‐C+H vs. Co_3_O_4_‐C were collected under a sequential increasing temperature from 100°C to 160°C with a 10°C step, as depicted in Figure 6e,f, respectively. Five Raman vibration peaks of Co_3_O_4_ were observed during the reaction. The F^1^_2g_ and E_2g_ modes correspond to O─Co─O bending in the tetrahedral position (Co^2+^‐O^2−^), while the other three modes are associated with Co─O stretching at the octahedral position (Co^3+^‐O^2−^). In contrast to the smooth curves of Co_3_O_4_‐C, the spectra of Co_3_O_4_‐C+H display notable fluctuations, reflecting its complex and interlaced high‐index facets. As the reaction temperature increases, the A_1g_ mode (Co─O) of Co_3_O_4_‐C+H shifts from 685 to 678 cm^−1^, and subsequently stabilizes at 680 cm^−1^. This trend indicates alterations in the Co─O bond within the Co^3+^‐O^2−^ structure, typically occurring when additional atoms are introduced into the lattice [42]. For this case, it is highly likely due to the incorporation of an oxygen atom resulting from the decomposition of N_2_O. Furthermore, according to Hooke's law [48], the Co─O bond force constant (*k*) in the Co^3+^‐O^2−^ structure was calculated (Figure S8). The result revealed that the *k* declined markedly during the reaction progress, suggesting that the *de*N_2_O reaction weakened the Co─O bond in Co_3_O_4_‐C+H. This confirms that the structure of Co^3+^‐O^2−^ was altered through its interaction with N_2_O. In contrast, the five peak positions of Co_3_O_4_‐C remained stable, although there was a decrease in peak intensity. This reduction in intensity might be due to the physical and chemical adsorption of N_2_O.

Furthermore, the electronic structure changes of the active phase of the Co_3_O_4_‐C+H catalyst during the *de*N_2_O process were investigated using in situ UV–vis diffuse reflectance spectroscopy (in situ UV–vis DRS). As illustrated in Figure 6g, the spectral signal of Co^3+^‐O^2−^undergoes a significant red‐shift as temperature increases, suggesting enhanced lattice vibrations and an increased Co─O atomic spacing. To rule out the influence of temperature, a controlled experiment was conducted under a He atmosphere (Figure S9). The results demonstrate that the redshift of the Co^3+^‐O^2−^ is more pronounced in a 7 vol.% N_2_O/He atmosphere (Figure 6g) compared to a pure He atmosphere. This strongly suggests an interaction between N_2_O and Co^3+^‐O^2−^ on the Co_3_O_4_‐C+H surface, which may alter the electron density distribution of Co^3+^‐O^2−^ [49]. Additionally, a reduction of bandgap was revealed as presented in Figure 6h, implying lower energy requirements for electron transitions, which is believed to be key in facilitating catalytic reaction [50].

These findings clearly indicate that Co^3+^ in Co_3_O_4_‐C+H is the crucial active site for *de*N_2_O reaction, which is very possibly the fundamental reason for its excellent *de*N_2_O performance at low temperature. Apparently, the Co^3+^ in Co_3_O_4_‐C+H, particularly those located on the interfacial (400−400) facet, offer unique properties that influence the entire *de*N_2_O process, encompassing both adsorption and the reaction pathway. These properties warrant thorough investigation.

### 2.5 Catalyst Construction Modeling and *de*N_2_O Simulation

A comparison of the single crystal facets (110), (400), and (311), as well as the interfacial facet (400–400) of Co_3_O_4_, was conducted using density functional theory (DFT) calculations to elucidate the origins of their *de*N_2_O activity.

**FIGURE 7:**
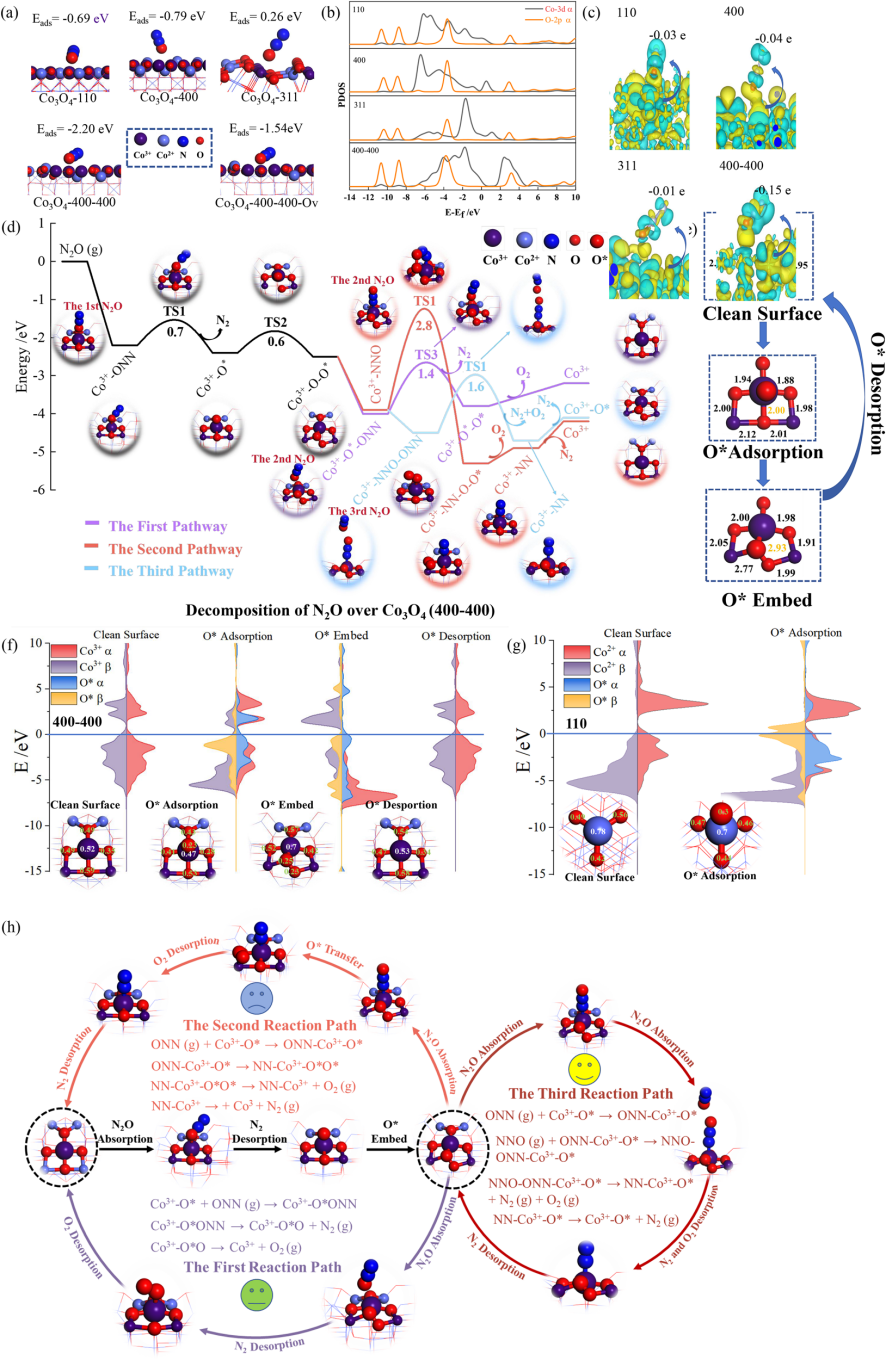
Study on the electronic structure characteristics of different crystal faces of Co_3_O_4_ Catalyst. (a) The adsorption energies of N_2_O on Co^3+^ active sites at different facets of Co_3_O_4_. (b) The partial density of states (PDOS) of N_2_O adsorption on Co^3+^ active sites at different facets of Co_3_O_4_. (c) The Mulliken charge and charge density difference (CDD) of N_2_O adsorption on Co^3+^ active sites at different facets of Co_3_O_4_, the cyan area represents electron deficiency and the yellow area represents electron enrichment. (d) The reaction energy diagrams of different N_2_O decomposition on the (400–400) facet. (e) The Co─O bond lengths of Co^3+^‐O^2−^ on the (400–400) facet at different structures. (f) The projected density of states (PDOS) and Mulliken charges of Co^3+^ and O^*^ at different structures on the (400–400) facet. (g) The projected density of states (PDOS) and Mulliken charges of Co^2+^ and O^*^ at different structures on the (110) facet. (h) The main reaction pathway network for N_2_O decomposition on the (400–400) facet.

Figures S10–S13 present the optimized configurations for N_2_O adsorption on these facets, specifically examining the adsorption of N_2_O molecules via −NNO or −ONN at the active sites of Co^3+^.The calculation results indicated that the O terminal of N_2_O is more readily adsorbed at the active sites on all facets and the minimum adsorption energies for each facet are shown in Figure 7a. The (400) crystal facet has the lowest adsorption energy at −0.79 eV, compared to (110) and (311); the Co^3+^ site on the (400–400) interface exhibits the lowest N_2_O adsorption energy at −2.2 eV, indicating high activity of Co^3+^ on high‐index interface facets. In addition, the presence of O vacancies was found not the most favorable site for N_2_O adsorption on the (400−400) facet, resulting in an adsorption energy of −1.54 eV. This is consistent with the experimental results of H_2_ or O_2_ pretreatment (Figure S14), thereby excluding the influence of Co^2+^ sites and O vacancies. These findings suggest that N_2_O adsorption is thermodynamically more favorable on Co^3+^ sites of the multi high‐index interfacial facet (400−400). Moreover, the partial density of states (PDOS), illustrated in Figure 7b, clearly shows a substantial overlap between the Co 3d and O 2p orbitals on the (400−400) facet. This indicates an electron transfer from the Co 3d orbitals to the O 2p orbitals, resulting in the formation of Co─O bonds. A detailed analysis of the charge density difference (CDD) (Figure 7c) reveals an increase in charge density for the Co─O bonds, while a decrease is observed for the N═O bonds. And Mulliken charge analysis also finds that the (400−400) surface transfers the highest charge (0.15 e) to N_2_O. These theoretical results demonstrate that Co^3+^ located at the interfacial facet (400−400) can enhance electron conduction efficiency, thereby improving electron transport to the O atom in N_2_O. This facilitates the activation and cleavage of N═O bonds. These findings adequately explain the previously observed low E_a_ of Co_3_O_4_‐C+H for *de*N_2_O.

Subsequently, we focused on the traditional (110) and high‐index (400−400) crystal facets, analyzing differences in their active sites (Co^3+^ and Co^2+^) and N_2_O decomposition pathways to reveal the effect of crystal facets on N_2_O decomposition. As shown in Figures S15 and S16, on the (110) facet, Co^3+^ exhibits a stronger adsorption energy (−2.12 eV) for the O terminal of N_2_O than Co^3+^; on the (400−400) facet, Co^2+^ shows weaker adsorption (−1.44 eV) compared to Co^3+^, which may be the key factor behind their differing catalytic activities. Based on these findings, detailed reaction pathway calculations were performed for Co^3+^ on the (400−400) facet and Co^2+^ on the (110) facet, with results presented in Figure 7d and Figure S17, respectively. The energy barrier for the breaking of the N═O bond in adsorbed N_2_O on the (400−400) facet is 0.7 eV, which is significantly lower than the 2.8 eV observed on the (110) facet. This finding underscores the critical role of Co^3+^ on the interfacial facet in facilitating the activation and cleavage of the N═O bond. As the reported mechanism for *de*N_2_O over the Co (110) facet [51], the O‐terminal of the second N_2_O molecule is directly adsorbed onto the Co^2+^‐O^*^ site on the (110) surface. This adsorption ultimately leads to the production of N_2_ and O_2_, with corresponding energy barriers of 2.1 and 0.5 eV, respectively. In addition, we investigated the stability of four oxygen vacancy (O_v_) configurations around Co^3+^ in the (400–400) model. As shown in Figure S18a, when the O_v_ is located on the left side of Co^3+^, the system energy is the lowest and the structure is the most stable. Based on this stable configuration, we calculated the adsorption energy of the N_2_O molecule adsorbed at the Co^3+^ site with the O end, which is −1.5 eV (Figure S18b), higher than −2.2 eV without O_v_. Meanwhile, the transition state energy barrier of its reaction path (Figure S18c) is 1.1 eV, also higher than 0.7 eV in the absence of O_v_, indicating that the presence of O_v_ weakens the catalytic activity of the Co^3+^ site for the decomposition of N_2_O. The adsorption behavior of N_2_O at the oxygen vacancy site was further calculated (Figure S18d), with an adsorption energy of −4.2 eV, which is significantly stronger than that at the Co^3+^ site, indicating that the oxygen vacancy is favorable for the initial adsorption and activation of N_2_O. However, the corresponding decomposition transition state energy barrier is as high as 2.8 eV, much higher than that at the Co^3+^ site, suggesting that the oxygen vacancy is not the main active center for the direct decomposition of N_2_O. Comprehensive analysis indicates that although the oxygen vacancy is not conducive to the activation of N_2_O at the Co^3+^ site, its presence can promote the migration and desorption of the O^*^ species generated from the decomposition of N_2_O, thereby indirectly promoting the overall reaction process. Therefore, the oxygen vacancy mainly plays an auxiliary role in the catalytic process, helping to alleviate the accumulation of surface oxygen and enhance the redox cycling ability of the catalyst.

On the interfacial facet (400−400), DFT calculations suggest that the oxygen terminal of the second N_2_O molecule cannot directly adsorb onto the Co^3+^‐O^*^ site (Figure S19). This result suggests that the O^*^ species would block the reaction process unless the second N_2_O can be adsorbed in another way. Considering the phenomenon of additional atoms introduction into the Co_3_O_4_‐C+H lattice confirmed by in situ Raman analysis (Figure 6e), we propose that an embedding of the O^*^ takes place prior to the adsorption of the second N_2_O molecule. Consequently, we evaluated the migration of O^*^ to four nearby oxygen sites adjacent to Co^3+^ to identify the most stable structure, as shown in Figure 7e and Figure S20. Upon recalculating the adsorption of N_2_O in the new configuration, it was surprising to find that both the O‐terminal and N‐terminal of the second N_2_O could be directly adsorbed at the embedded O^*^ site and the Co^3+^ site, with adsorption energies of −1.5 and −1.4 eV, respectively (Figure S21). The adsorption energy of N_2_O on the N terminal of Co^3+^ in the new structure (−1.4 eV) is stronger than that in the original structure (−1.0 eV). This aligns with the Mullikan charge analysis, showing more charge transfer to N_2_O on the new motif (Figure S22). In other words, there are two potential reaction pathways over the facet (400−400). The first is that the O‐terminal of the second N_2_O adsorbed onto the embedded O^*^ site, leading to the formation of N_2_ and O_2_ with energy barriers of 1.4 and 0.3 eV, respectively. The second way is that the N‐terminal adsorbed onto the Co^3+^ site, which necessitates overcoming a higher energy barrier of 2.8 eV to produce O_2_ and N_2_. Clearly, the first pathway is more favorable for the *de*N_2_O process.

Noteworthily, whether the first or the second pathway, the Co^3+^ site on the (400−400) facet undergoes a complete cycle from N_2_O adsorption to O^*^ desorption. Nevertheless, the Co_3_O_4_‐C+H catalyst, examined post‐reaction, shows an increase in lattice oxygen content without significant alterations in the Co^3+^ sites (Figure S23). These findings suggest that the O^*^ species remain within the lattice. In light of this contradiction, we refocused our attention on the O^*^ embedded Co^3+^ motif. Upon further examination of this structure, DFT calculations find that the Co^3+^‐O^*^ motif exhibits a strong adsorption capacity for N_2_O after adsorption of the second N_2_O by N‐terminal. Thus, an alternative reaction pathway is proposed. Initially, the N‐terminal of the second N_2_O adsorbs onto the Co^3+^ site. Subsequently, the O‐terminal of the third N_2_O binds to the O‐terminal of the second N_2_O, forming an t*rans*‐N_2_O_2_^2−^ intermediate by overcoming an energy barrier of 1.6 eV. Interestingly, this finding aligns precisely with the t*rans*‐N_2_O_2_^2−^ intermediates observed through in situ DRIFTS spectroscopy. Subsequently, the t*rans*‐N_2_O_2_^2−^ decomposes into N_2_ and O_2_, and ultimately, N_2_ desorbs from the Co^3+^ site with an energy barrier of 0.6 eV. This new pathway demonstrates an energy barrier of 1.6 eV, comparable to the first pathway's 1.4 eV, indicating that the *de*N_2_O process via this new route is plausible over the (400−400) facet and cannot be disregarded. More importantly, this pathway preserves the integrity of the Co^3+^‐O^*^ motif, thereby confirming the feasibility of this route.

Furthermore, the electronic structure of the Co^3+^‐O^*^ site on the (400−400) facet was analyzed in conjunction with the PDOS and Mulliken charge analysis to clarify the reasons for its high catalytic activity, as illustrated in Figure 7f. Compared with the Co^3+^ on clean surface and O^*^ adsorption structure, the PDOS of Co^3+^ in the O^*^ embedded structure shifts toward the Fermi level. This shift indicates that Co^3+^ is more likely to lose electrons during the reaction, thereby facilitating the electron transferring during the catalytic reaction. The Mulliken charge analysis reveals that Co^3+^ in this new structure has the highest capacity for electron loss, measured at 0.7e. This finding underscores its unique electronic structure, which fundamentally contributes to its efficient adsorption on the N‐terminal of N_2_O. Additionally, a comparison of the adsorption of O^*^ on Co^3+^ in the (400−400) facet with that on Co^2+^ in the (110) facet reveals that PDOS for O^*^ on the (110) facet peaks closer to the Fermi level (Figure 7g), indicating that the second N_2_O molecule is more likely to react with O^*^ on the (110) facet. However, the more substantial overlap of O 2p and Co 3d orbitals on the (110) facet, compared to the (400−400) facet, indicates a more stable Co─O^*^ bond. This results in higher fracture energy and the inhibition of *de*N_2_O at low temperatures. Figure 7h depicts the network of mechanisms involving the three reaction pathways for *de*N_2_O on the interfacial facet (400−400). The active Co^3+^‐O^*^ motif was formed by O^*^ embedding into the adjacent lattice from N_2_O decomposition on the Co^3+^. This active motif not only exhibits significantly lower energy barrier for the conventional *de*N_2_O pathway, but also offers an alternative route for the rapid *de*N_2_O. Moreover, the newly identified pathway shortens the reaction steps and enhances the efficiency of *de*N_2_O. This enhancement aligns with the catalyst's high TOF and its outstanding low‐temperature activity, as observed in the Co_3_O_4_‐C+H catalyst.

## 3 Conclusion

In summary, we proposed a simple two‐step strategy, involving co‐precipitation followed by hydrothermal treatment, to effectively synthesize Co_3_O_4_ (Co_3_O_4_‐C+H) featuring high‐index facets (400) and (400), specifically the interfacial facet (400−400). The specific interfacial facet (400−400) enhances the *de*N_2_O performance of Co_3_O_4_‐C+H by providing numerous Co^3+^ active sites, more reactive Co─O bonds, and improved capabilities in electron and O^*^ transfer. Our results indicate that Co_3_O_4_‐C+H achieved excellent performance in *de*N_2_O at low temperature (300°C) with conversion efficiency of up to 99% and TOF of 1.86 × 10^−2^ s^−1^, which significantly surpasses that of Co_3_O_4_‐C, which achieved only an 13% conversion efficiency and a TOF of 3.87 × 10^−4^ s^−1^. Furthermore, the structural evolution of Co^3+^ on Co_3_O_4_‐C+H upon reaction progress has been systematically investigated using a series of in situ techniques. And the results reveal that (400−400) interfacial facet could weaken the Co─O bond and facilitate electron transfer to the O‐terminal of N_2_O, thereby enhance the *de*N_2_O reaction. Theoretical calculations also confirm that Co^3+^ on the interfacial facet (400−400) can enhance electron conduction efficiency and quantitively indicate a barrier of only 0.7 eV for the cleavage of N═O bonds on the (400−400) facet, compared to a 2.8 eV barrier on the Co_3_O_4_ (110) facet.

More interestingly, combining with theoretical calculations, we have identified a novel active Co^3+^‐O^*^ motif. This motif is formed when the O^*^ species, generated from N_2_O decomposition, incorporates into the lattice. In addition, the newly formed active Co^3+^‐O^*^ motif displays a distinctive electronic structure characterized by improved electron transfer to the N‐terminal of N_2_O, which significantly enhances its adsorption efficiency at the N‐terminal of N_2_O, and this provides an alternative pathway for the reduction of N_2_O. In this proposed mechanism, the N‐terminal of a second N_2_O molecule adsorbs onto the Co^3+^ site, while the O‐terminal of a third N_2_O molecule binds to the O‐terminal of the second N_2_O. This sequence results in the formation of an t*rans*‐N_2_O_2_^2−^ intermediate, which subsequently decomposes directly into N_2_ and O_2_. This new pathway shortens the reaction steps and enhances the efficiency of *de*N_2_O.

This study presents a simple method to synthesize multi high‐index crystal facets catalysts and verifies the mechanism of low‐temperature *de*N_2_O catalysis through systematic experiments and theoretical calculations. It provides a crucial theoretical foundation for developing catalysts that activate inert molecules at low temperatures, facilitating their activation and utilization.

## Conflicts of Interest

The authors declare no conflict of interest.

## Supporting information




**Supporting file**: advs74735‐sup‐0001‐SuppMat.docx

## Data Availability

The data supporting the findings of this study are available in the article and supplemental information or from the corresponding authors upon reasonable request.
