# Supplementary material for: In Situ Elucidations of the Mechanism for Inert Molecular N2O Decomposition at Low Temperature Catalyzed by High‐Index Interfacial Exposed Co3O4
Source: Adv Sci (Weinh). 2026 Mar 9;13(28):e74735. doi: 10.1002/advs.74735 (PMC13185883; doi:10.1002/advs.74735)
Supplement: Supplementary file 1 — Supporting file: advs74735‐sup‐0001‐SuppMat.docx [file ADVS-13-e74735-s001.docx]

**Supporting Information**

***In-situ* elucidations of the mechanism for inert molecular N_2_O decomposition at low-temperature catalyzed by high-index interfacial exposed Co_3_O_4_**

Lei Pan,^1^ Qian Mi,^1^ Minghao Wang,^2^ Luyu Wang, ^1^ Yiyang Xie,^3^ Huanjie Zhang,^1^ Ruinian Xu,^1,^* and Biaohua Chen ^1^

1 College of Environmental Science and Engineering, Beijing University of Technology, Beijing 100124, China

2 Material Science and Engineering, Beijing University of Technology, Beijing 100124, China

3 Key Laboratory of Optoelectronics Technology, Ministry of Education, Beijing University of Technology, Beijing 100124, China

*Corresponding author: xuruinian@bjut.edu.cn

**Table of Contents**

[**Experimental Procedures** 4](#_Toc221280965)

[**Materials** 4](#_Toc221280966)

[Synthesis of Co_3_O_4_-C catalyst 4](#_Toc221280967)

[Synthesis of Co_3_O_4_-H catalyst 4](#_Toc221280968)

[Synthesis of Co_3_O_4_-C+H catalyst 4](#_Toc221280969)

[**Characterization** 5](#_Toc221280970)

[**Catalytic activity evaluation and kinetic tests** 6](#_Toc221280971)

[**Density functional theory (DFT) calculations** 8](#_Toc221280972)

[**Results and Discussion** 9](#_Toc221280973)

[**Supplementary Figures** 9](#_Toc221280974)

[Figure S1. The N_2_ selectivity of Co_3_O_4_-C, Co_3_O_4_-H, and Co_3_O_4_-C+H catalysts. 9](#_Toc221280975)

[Figure S2. Effects of different synthesis conditions on the properties of Co_3_O_4_-C+H under reaction conditions of 7 vol.% N_2_O, 2 vol.% H_2_O, with He as the balance gas and a GHSV of 10,000 h^-1^. a) Hydrothermal reaction temperature. b) Hydrothermal reaction time. c) Total volume of precipitant drops added. d) Rate of precipitant drop addition. 10](#_Toc221280976)

[Figure S3. a,b) The effect of H_2_O and O_2_ on the properties of Co_3_O_4_-C+H catalyst. 11](#_Toc221280977)

[Figure S4. SEM images of Co_3_O_4_-C+H catalysts. a) Co_3_O_4_-C+H, 60 mL/h, pH=8.5. b) Co_3_O_4_-C+H, 60 mL/h, pH=9.5. c) Co_3_O_4_-C+H, 60 mL/h, pH=10.15. d) Co_3_O_4_-C+H, 30 mL/h, pH=9.5. e) Co_3_O_4_-C+H, 60+120 mL/h, pH=9.5. 12](#_Toc221280978)

[Figure S5. a,b) The XPS spectra of Co 2p and O 1s of Co_3_O_4_-C+H catalysts prepared at different pH values under a Na_2_CO_3_ flow rate of 60 mL/h. 13](#_Toc221280979)

[Figure S6. Kinetic tests were conducted on the synthesized Co_3_O_4_ catalyst under high gas hourly space velocity and high concentration of N_2_O conditions. a) The N_2_O conversion of Co_3_O_4_-C catalyst. b) The N_2_O conversion of Co_3_O_4_-H catalyst. c) The N_2_O conversion of Co_3_O_4_-C+H catalyst. 14](#_Toc221280980)

[Figure S7. *In-situ* DRIFTS of Co_3_O_4_-C, Co_3_O_4_-H and Co_3_O_4_-C+H catalysts at 250^o^C in a 7 vol.% N_2_O/He atmosphere. 15](#_Toc221280981)

[Figure S8. The Co-O bond force constant (k) in the Co^3+^-O^2-^ structure at different temperatures. 16](#_Toc221280982)

[Figure S9. In-situ UV-vis DRS for Co_3_O_4_-C+H catalyst under different conditions. a) 7 vol.% N_2_O/He atmosphere. b) Pure He atmosphere. c) Curves under two atmospheres at 250^o^C. 17](#_Toc221280983)

[Figure S10. The adsorption energies of N_2_O on Co^3+^ active sites at (110) facet of Co_3_O_4._ a) The top view of the 110 crystal facet of Co_3_O_4_. b) The O-terminal of N_2_O is adsorbed at the Co^3+^ site. c) The N-terminal of N_2_O is adsorbed at the Co^3+^ site. 18](#_Toc221280984)

[Figure S11. The adsorption energies of N_2_O on Co^3+^ active sites at (400) facet of Co_3_O_4._ a) The top view of the (400) crystal facet of Co_3_O_4_. b) The O-terminal of N_2_O is adsorbed at the Co^3+^ site. c) The N-terminal of N_2_O is adsorbed at the Co^3+^ site. 19](#_Toc221280985)

[Figure S12. The adsorption energies of N_2_O on Co^3+^ active sites at (311) facet of Co_3_O_4._ a) The top view of the (311) crystal facet of Co_3_O_4_. b) The O-terminal of N_2_O is adsorbed at the Co^3+^ site. c) The N-terminal of N_2_O is adsorbed at the Co^3+^ site. 20](#_Toc221280986)

[Figure S13. The adsorption energies of N_2_O on Co^3+^ active sites at (400-400) facet of Co_3_O_4._ a) The top view of the 400-400 composite crystal facet of Co_3_O_4_. b) The O-terminal of N_2_O is adsorbed at the Co^3+^ site on the interface. c) The N-terminal of N_2_O is adsorbed at the Co^3+^ site on the interface. 21](#_Toc221280987)

[Figure S14. Effect of different atmosphere pretreatment on the performance of Co_3_O_4_-C+H catalyst. 22](#_Toc221280988)

[Figure S15. The adsorption energies of N_2_O on Co^2+^ active sites at (110) facet of Co_3_O_4._ a) The O-terminal of N_2_O is adsorbed at the Co^2+^ site. b) The N-terminal of N_2_O is adsorbed at the Co^2+^ site. 23](#_Toc221280989)

[Figure S16. The adsorption energies of N_2_O on Co^2+^ active sites at (400-400) facet of Co_3_O_4._ a) The O-terminal of N_2_O is adsorbed at the Co^2+^ site. b) The N-terminal of N_2_O is adsorbed at the Co^2+^ site. 24](#_Toc221280990)

[Figure S17. The reaction energy diagrams of different N_2_O decomposition on the (110) facet. 25](#_Toc221280991)

[Figure S18. a) 400-400 structure containing O_v_. b) Adsorption energy of the O end of N_2_O on Co^3+^ sites containing O_v_. c) Reaction path of N_2_O on Co^3+^ sites containing O_v_. d) Adsorption energy of the O end of N_2_O on O_v_. e) Reaction path of N_2_O at O_v_. 26](#_Toc221280992)

[Figure S19. The adsorption energies of the second N_2_O on Co^3+-^O* active sites at (400-400) facet of Co_3_O_4._ 27](#_Toc221280993)

[Figure S20. The decomposed O* of the first N_2_O is transferred to the four adjacent oxygens next to Co^3+^. a) Left. b) Right. c) Front. d) Rear. 28](#_Toc221280994)

[Figure S21. The adsorption energies of the second N_2_O on new Co^3+-^O* active sites at (400-400) facet of Co_3_O_4._ a) The O-terminal of the second N_2_O is adsorbed at the O* site on the interface. b) The N-terminal of the second N_2_O is adsorbed at the O* site on the interface. c) The O-terminal of the second N_2_O is adsorbed at the Co^3+^ site on the interface. d) The N-terminal of the second N_2_O is adsorbed at the Co^3+^ site on the interface. 29](#_Toc221280995)

[Figure S22. The charge density difference (CDD) of N_2_O adsorption on different structures of Co_3_O_4_(400-400), the cyan area represents electron deficiency and the yellow area represents electron enrichment. a) The N-terminal of the first N_2_O is adsorbed at the Co^3+^ site on the interface. b) The N-terminal of the second N_2_O is adsorbed at the Co^3+^ site on the new active Co^3+^-O* motif. 30](#_Toc221280996)

[Figure S23. The XPS spectra of O 1s of Co_3_O_4_-C+H catalyst after reaction. 31](#_Toc221280997)

[**Supplementary Tables** 32](#_Toc221280998)

[Table S1. The T_90_ of different catalysts reported in the literatures. 32](#_Toc221280999)

[Table S2. BET for Co_3_O_4_-C, Co_3_O_4_-H, Co_3_O_4_-C+H catalysts. 33](#_Toc221281000)

[Table S3. Kinetic parameters of N_2_O catalytic decomposition over Co_3_O_4_ catalysts.. 34](#_Toc221281001)

[**References** 35](#_Toc221281002)

**Experimental Procedures**

**Materials**

**Synthesis of Co_3_O_4_-C catalyst**

Synthesis by co-precipitation method: Dissolve 10 g of cobalt nitrate (Co(NO_3_)_2_·6H_2_O) in 50 ml of deionized water and stir it in a constant temperature water bath at 40^o^C for 30 minutes. Add a solution of sodium carbonate (0.5 mol/L, 60 mL/h) to adjust the pH value of the reaction solution to 9, and then stir for 3 hours. Clean the obtained reaction liquid with deionized water until neutral, then dry it in an oven at 120^o^C for 5 hours. Grind the sample into powder, place it in a magnetic boat, and calcine it at 500^o^C for 3 hours in a Muffle furnace. Finally, let it cool naturally to room temperature to obtain Co_3_O_4_-C catalyst.

**Synthesis of Co_3_O_4_-H catalyst**

Hydrothermal synthesis: Dissolve 10g of cobalt nitrate (Co(NO_3_)_2_·6H_2_O) in 50 ml of deionized water and add a solution of sodium carbonate (0.5 mol/L). Adjust the pH value to 9. Place the mixture in a reactor and conduct hydrothermal reaction at 180^o^C with stirring at 400 r/min for 3 hours. Clean the obtained reaction liquid with deionized water until neutral, then dry it in an oven at 120^o^C for 5 hours. Grind the sample into powder, transfer it to a magnetic boat, and calcine it in a Muffle furnace at 500^o^C for 3 hours. Finally, let it cool naturally to room temperature to obtain Co_3_O_4_-H catalyst.

**Synthesis of Co_3_O_4_-C+H catalyst**

For co-precipitation + hydrothermal two-step synthesis, dissolve 10g of cobalt nitrate hexahydrate in 50 ml of deionized water and stir it in a constant temperature water bath at 40^o^C for 30 minutes. Add a solution of sodium carbonate (0.5 mol/L, 60 mL/h) to adjust the pH value of the reaction solution to 9. Place the mixture in a reactor and conduct hydrothermal reaction at 180^o^C with stirring at 400 r/min for 3 hours. Clean the resulting reaction liquid with deionized water until neutral, then dry it in an oven at 120^o^C for 5 hours. Grind the sample into powder and place it in a magnetic boat, followed by calcination in a Muffle furnace at 500^o^C for 3 hours. Finally, let it cool naturally to room temperature to obtain Co_3_O_4_-C+H catalyst. The Co_3_O_4_-C+H catalysts prepared under other different working conditions all followed the same synthesis process, with only the target variables being adjusted accordingly to systematically optimize and determine the best synthesis conditions, including hydrothermal time, hydrothermal temperature, the amount and rate of addition of the precipitating agent.

**Characterization**

The specific surface area and pore structure parameters of the as-prepared catalysts were determined through Brunauer-Emmett-Teller (BET) method and Barrett-Jioner-Halenda (BJH) method by using N_2_ adsorption/desorption analyzer (Nova2200E, Quantachrome, USA) at 77 K. The phase composition of the as-prepared catalysts was determined by X-ray diffraction (XRD, D8 Advance, Germany). The crystal phase structure of cobalt in the catalyst was characterized by Raman spectroscopy (LabRAM HR Evolution, HORBIBA, France). The valence states of related elements in the catalysts were detected by X-ray photoelectron spectroscopy (XPS, Escalab 250Xi, USA). Field emission scanning electron microscopy (FESEM, Zeiss, Sigma 500, Germany) was used to characterize the surface morphology of the catalysts. The element distribution on the surface of the catalysts was determined by energy dispersive X-ray spectrometry (EDS/Mapping, EDS Quantax, Bruker, Germany). Electron paramagnetic resonance (EPR, Bruker A300, Germany) was used to characterize the oxygen vacancy of catalysts. The oxidation state of Co was detected by ultraviolet–visible spectroscopy (Lambda 850 UV/Vis Spectrometer, PerkinElmer, USA).

Temperature-programmed reduction (TPR) experiments were tested on chemisorbed instrument (AutoChem 2920, Micromeritics, USA) instrument to detect the reduction of valence state of Co. Before the experiments, the 0.02 g catalyst was treated at 550^o^C for 1 h in a flow of 30 mL min^-1^ He (> 99.999%) atmosphere. After cooling down to 50^o^C, the catalyst was exposed to H_2_ (5%, 30 mL/min) for 1 h. Afterwards, the catalyst was heated in H_2_ flow (5%, 30 mL/min) from 50-900^o^C at a rate of 10^o^C/min and the signal of H_2_ was detected by chemisorbed instrument.

Temperature-programmed desorption (TPD) experiments were performed on mass spectrometer (QMS 403 QUADRO, NETZSCH, Germany) instrument to detect the desorption amount of O_2_ and N_2_O. Before the experiments, the 0.03 g catalyst was treated at 550^o^C for 1 h in a flow of 30 mL/min He (> 99.999%) atmosphere. After cooling down to 50^o^C, the catalyst was exposed to O_2_ (> 99.999%, 30 mL/min) or N_2_O (> 99.999%, 30 mL/min) for 1 h and then purged with He (> 99.999%, 30 mL/min). Afterwards, the catalyst was heated in He flow (> 99.999%, 30 mL/min) from 50-550^o^C at a rate of 10^o^C/min and the signal of O_2_ and N_2_O was detected by mass spectrometer.

The temperature programmed surface reaction (TPSR) experiment was conducted over a fixed-bed reactor system being connected with a mass spectrometer (QMS 403 QUADRO, NETZSCH, Germany). Initially, the catalyst was pretreated by He (> 99.999%, 30 mL/min) for 1 h at 550^o^C; then the gas mixture of 7 vol.% N_2_O/He was introduced into the reactor being heated at a rate of 5^o^C/min; simultaneously, the corresponding M/e signals were recorded by the mass spectrometer with multiple ion detection (MID), which includes 44 (N_2_O), 32 (O_2_), 28 (N_2_), 16 (O), 30 (NO), 46 (NO_2_), 62 (NO_3_) and 60 (N_2_O_2_).

*In-situ* Diffuse reflaxions infrared fourier transformations spectroscopy (DRIFTS) experiments were conducted on FTIR spectrometer (Tensor Ⅱ, Bruker, Germany) with BaF_2_ windows, which is equipped with liquid nitrogen cooled high-sensitive MCT detector. Prior to each experiment, the samples were pretreated at 550^o^C for 1 h in a flow of 50 mL/min He (> 99.999%) and then cooled to 200^o^C. In the cooling process, the spectra of catalyst at 350, 300, 250 and 200^o^C were collected in following He (> 99.999%, 50 mL/min) and was subtracted from the sample spectrum. Then the mixture of 7 vol.% N_2_O/He was introduced to react with catalysts. Subsequently, the spectra were recorded from 200^o^C to 350^o^C and the corresponding spectra were collected with reaction times for 0.5 h reaction. All spectra were recorded from 4000 to 400 cm^-1^ by accumulating 32 scans with a resolution of 4 cm^-1^.

*In-situ* Raman experiments were conducted on Raman spectrometer (LabRAM HR Evolution, HORBIBA, France) with BaF_2_ windows, which is equipped with a laser source with a wavelength of 532 nm and a power of 25 mW. Prior to each experiment, the samples were pretreated at 550^o^C for 1 h in a flow of 50 mL/min He (> 99.999%) and then cooled to 100^o^C. Then the mixture of 7 vol.% N_2_O/He was introduced to react with catalysts. Subsequently, the spectra were recorded from 100^o^C to 160^o^C and the corresponding spectra were recorded from 1000 to 100 cm^-1^.

*In-situ* Ultraviolet-visible diffuse reflectance spectroscopy (UV-Vis DRS) experiments were conducted on UV spectrometer (Lambda 850+, PerkinElmer, America) with BaF_2_ windows, and BaSO_4_ was used as the baseline for zeroing. Prior to each experiment, the samples were pretreated at 550^o^C for 1 h in a flow of 50 mL/min He (> 99.999%) and then cooled to 20^o^C. Then the mixture of 7 vol.% N_2_O/He was introduced to react with catalysts. Subsequently, the spectra were recorded from 20^o^C to 250^o^C and the corresponding spectra were recorded from 800 to 250 nm.

**Catalytic activity evaluation and kinetic tests**

The performance of Co_3_O_4_ catalysts for eliminating N_2_O was carried in a fixed-bed reactor with a quartz tube of 0.8 cm (ID) × 45 cm. Prior to conducting catalytic activity evaluation tests, catalysts were pretreated in a flow of He (50 mL/min) at 550^o^C for 1 h. During the test, 0.60 g catalyst was loaden into the reactor, corresponding to a gas hourly space velocities (GHSV) of 10,000 h^-1^. The total flow rate of simulated flue gas is 50 mL/min, whose components contain 7 vol.% N_2_O (> 99.999%), 0-10 vol.% O_2_ (> 99.999%) and balance gas He. The concentrations of each component (N_2_O, O_2_, N_2_, NO and NO_2_) were detected by gas chromatography (Shimadzu GC2030, Japan). The conversion efficiency of N_2_O and selectivity of N_2_ were calculated by the following equation:

| $N_{2}O\text{ }C\text{onversion = (}\frac{N_{2}O_{\text{in}}\text{ - N}_{2}O_{out}}{N_{2}O_{\text{in}}}\text{) }\times\text{ 100\%}$ | (S1) |
| --- | --- |
| $N_{2}\text{ Selectivity = (}\frac{2N_{2out}}{2N_{2out}\text{ + NO}_{2out}\text{ + NO}_{out}}\text{) }\times\text{ 100\%}$ | (S2) |

In which, *N_2_O*_in_ and *N_2_O*_out_ are N_2_O concentration at the inlet and outlet of the reactor, ppm; *NO*_out_ and *NO_2_*_out_ are NO and NO_2_ concentration at the inlet and outlet of the reactor, respectively.

For kinetic tests, the N_2_O decomposition rates of Co_3_O_4_-C and Co_3_O_4_-C+H were measured under steady states at different reaction conditions. Generally, 50-100 mg of catalysts were pretreated in a flow of He (50 mL/min) at 550^o^C for 1 h and then cooled to room temperature for further kinetic evaluation. As published paper,^1-4^ N_2_O catalytic decomposition is a first-order kinetic reaction, rate constant (*k*, m^3^⋅s^-1^⋅g^-1^), apparent activation energy (*E_a_*, kJ⋅mol^-1^), and reaction rate (*r*, μmol⋅s^-1^⋅g^-1^) for N_2_O decomposition were calculated by below Eqs. (S3-6).^5,6^ *F_0_* is the N_2_O flow rate over the reactor (mol⋅s^-1^); *W_cat_* is catalyst mass (g); *A* is the pre-exponential factor; *R* is the gas constant (8.3145 J⋅mol^-1^⋅K^-1^). TOF (s^-1^) is defined as the moles of N_2_O molecules converted per second on the moles of Co^3+^ atoms with Co_3_O_4_ catalysts, and *n* (μmol⋅g^-1^) is the active site amount, detected by H_2_-TPR. Note that The N_2_O conversion was strictly controlled below 10% to ensure a differential reactor, where the diffusion and heat transfer effects could be ignored.

| $k=\text{ }\frac{F_{0}\ln(1-C_{N_{2}O})}{N_{2}O_{in}W_{cat}}$ | (S3) |
| --- | --- |
| $k=\text{ Aexp(}-\frac{E_{a}}{RT}\text{)}$ | (S4) |
| $r=\text{ k}N_{2}O_{in}(1-C_{N_{2}O})$ | (S5) |
| $TOF=\text{ }\frac{r}{n}$ | (S6) |

**Density functional theory (DFT) calculations**

The density functional theory (DFT) implementation in CP2K/Quickstep is based on a hybrid Gaussian plane wave (GPW) scheme.^7-11^ A matrix orbital transformation procedure was used for the wave function optimization and self-consistent field (SCF) convergence standard is 10^-7^ eV. The EPS_DEFAULT 1.0E-14, the Max step size 0.003, RMS step size 0.0015, Max gradient 0.0006, RMS gradient 0.0003.^12^

According to XRD characterization and literature reports, the Co_3_O_4_ cell was selected as Fd3m mp-18748 and then optimized with a K value of 5*5*5. Based on TEM characterization results, crystal planes Co_3_O_4_-110, Co_3_O_4_-400, and Co_3_O_4_-311 were cut along with interfacial plane C_3_O_4_-(400−400) using the optimized surfaces. A vacuum layer of 15 Å was applied, positioned 3 Å away from the bottom, while the lower four layers remained fixed. The adsorption distance of N_2_O on the surface was set at 1.5 Å. The adsorption calculation focused separately on Co^2+^ and Co^3+^ sites at both O and N terminals of N_2_O on different crystal planes. The 2s, 2p electrons of N and O, 3d, 4s, 2p electrons of Co were treated as valence, and U = 4 eV for Co 3d was chosen in the catalytic systems.^13-15^ The rest core electrons were represented by Goedecker-Teter-Hutter (GTH) pseudopotentials.^16-17^ The Gaussian basis set was double-ς with one set of polarization functions (DZVP),^18^ and the plane wave cutoff was set to 550 Ry. Perdew-Burke-Ernzerhof (PBE) functional was used to describe the exchange-correlation effects.^19^ In static calculations, the geometries were optimized by Broyden-Fletcher-Goldfarb-Shanno (BFGS) minimizer. Note that due to the large size of the large size of the supercells, only Г point was used in all calculations. To obtain the transition states and reaction pathway, the climbing image nudged elastic band (CI-NEB) method was used.^20,21^ The adsorption energy (E_ads_) of reactants of reactants or reaction intermediates was calculated as following:^22^

| $E_{\text{ads}}\text{ = E}_{sys}\text{ - (E}_{abs}\text{ + E}_{cat})$ | (S7) |
| --- | --- |

where E_sys_ was the total energy of the adsorption system, E_abs_ was the energy of adsorbates, and E_cat_ was the energy of the catalyst, respectively.

# Results and Discussion

# Supplementary Figures

## Figure S1. The N_2_ selectivity of Co_3_O_4_-C, Co_3_O_4_-H, and Co_3_O_4_-C+H catalysts.


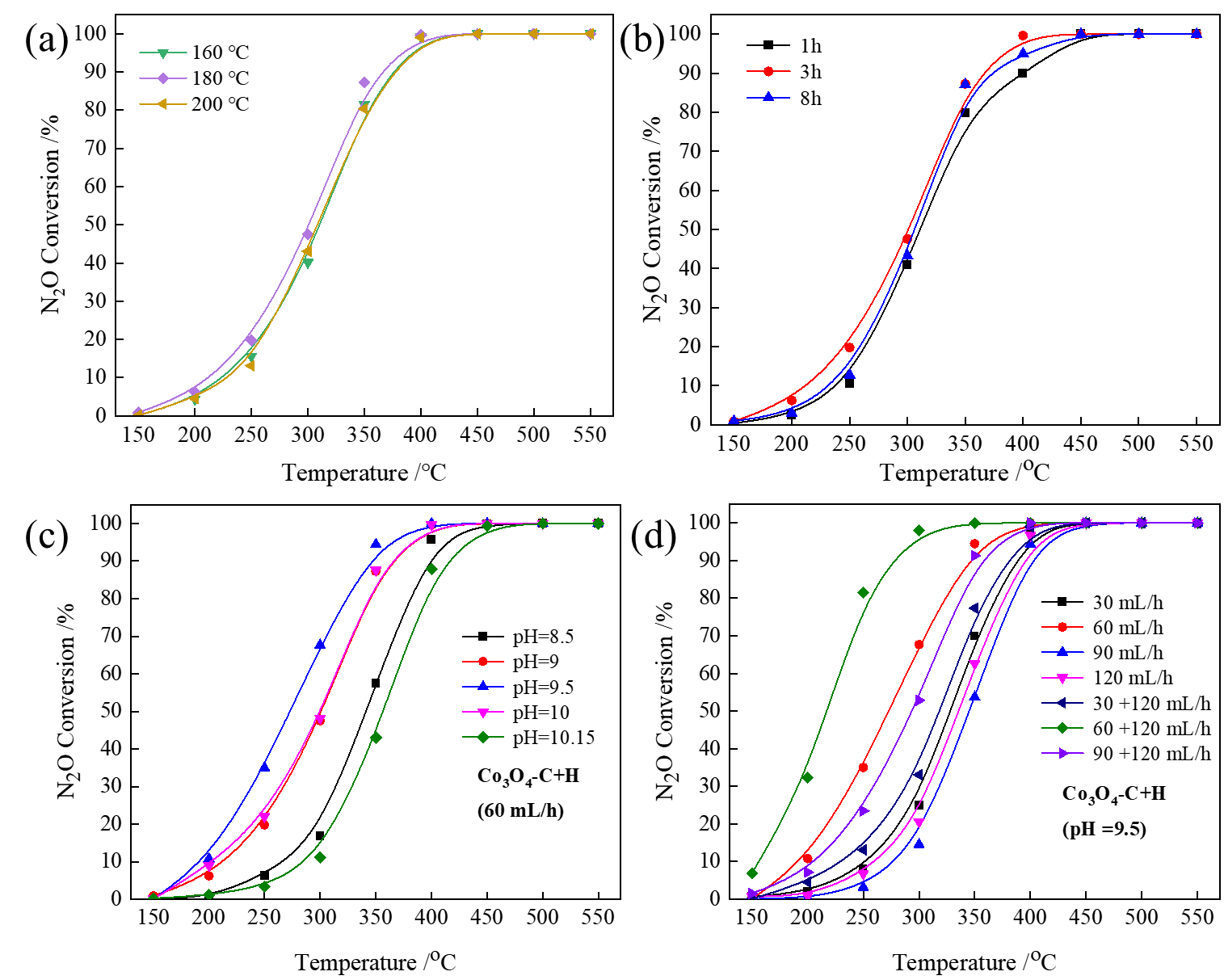


## Figure S2. Effects of different synthesis conditions on the properties of Co_3_O_4_-C+H under reaction conditions of 7 vol.% N_2_O, 2 vol.% H_2_O, with He as the balance gas and a GHSV of 10,000 h^-1^. a) Hydrothermal reaction temperature. b) Hydrothermal reaction time. c) Total volume of precipitant drops added. d) Rate of precipitant drop addition.


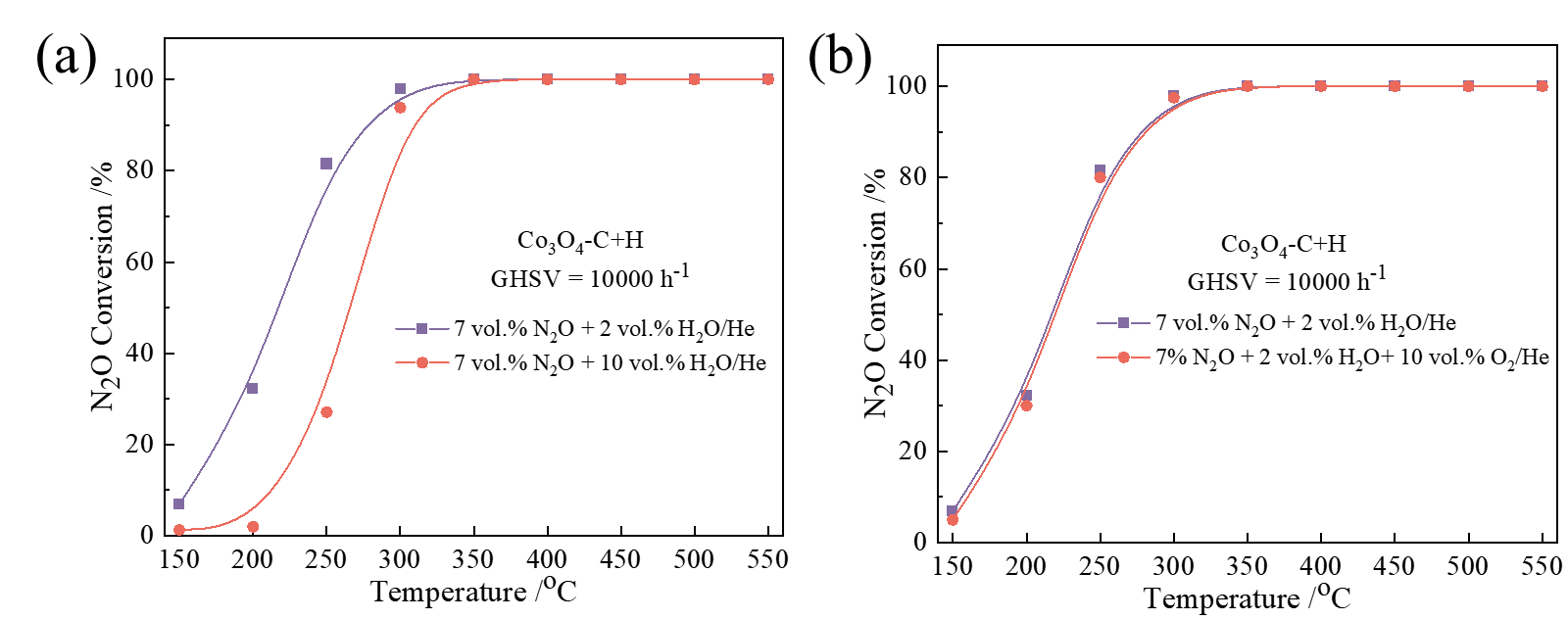


## Figure S3. a,b) The effect of H_2_O and O_2_ on the properties of Co_3_O_4_-C+H catalyst.


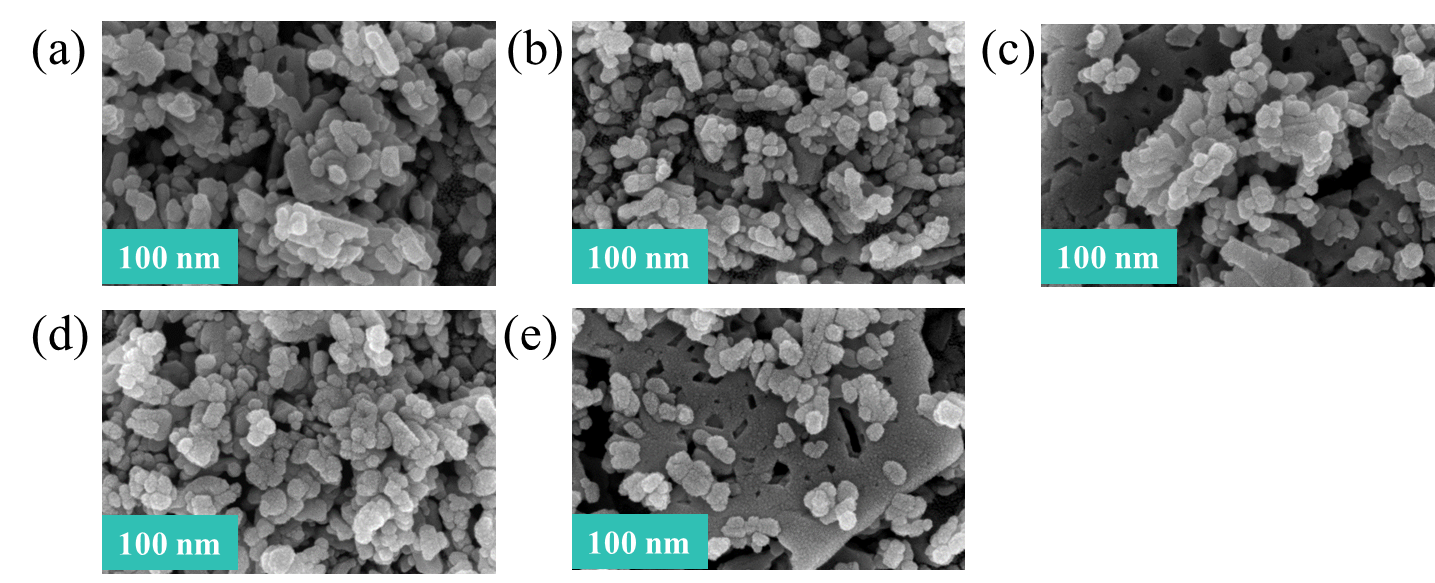


## Figure S4. SEM images of Co_3_O_4_-C+H catalysts. a) Co_3_O_4_-C+H, 60 mL/h, pH=8.5. b) Co_3_O_4_-C+H, 60 mL/h, pH=9.5. c) Co_3_O_4_-C+H, 60 mL/h, pH=10.15. d) Co_3_O_4_-C+H, 30 mL/h, pH=9.5. e) Co_3_O_4_-C+H, 60+120 mL/h, pH=9.5.


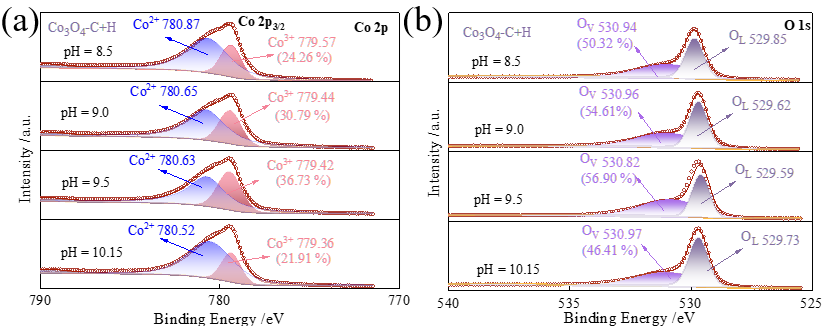


## Figure S5. a,b) The XPS spectra of Co 2p and O 1s of Co_3_O_4_-C+H catalysts prepared at different pH values under a Na_2_CO_3_ flow rate of 60 mL/h.


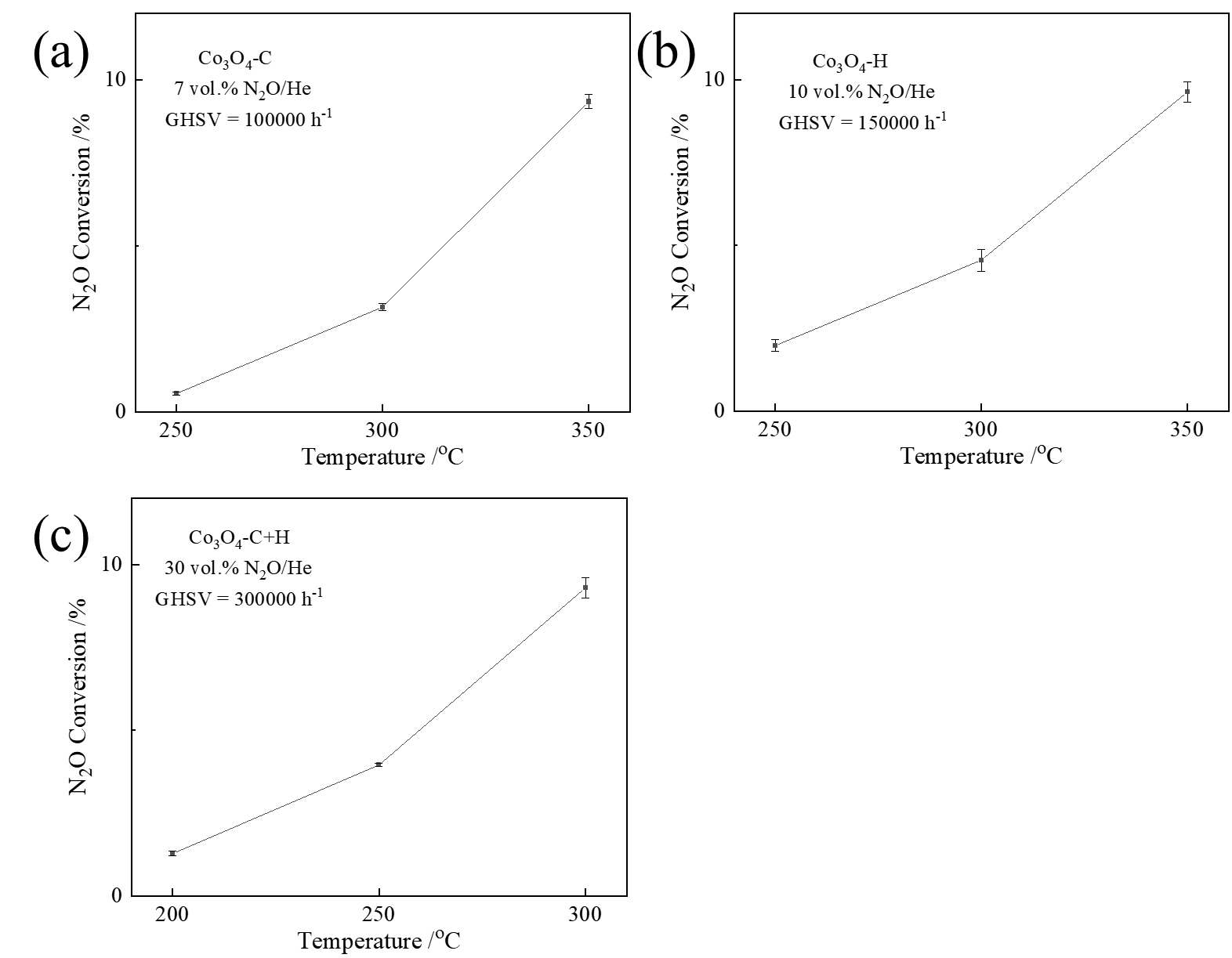


## Figure S6. Kinetic tests were conducted on the synthesized Co_3_O_4_ catalyst under high gas hourly space velocity and high concentration of N_2_O conditions. a) The N_2_O conversion of Co_3_O_4_-C catalyst. b) The N_2_O conversion of Co_3_O_4_-H catalyst. c) The N_2_O conversion of Co_3_O_4_-C+H catalyst.

## Figure S7. *In-situ* DRIFTS of Co_3_O_4_-C, Co_3_O_4_-H and Co_3_O_4_-C+H catalysts at 250^o^C in a 7 vol.% N_2_O/He atmosphere.

Figure S8. The Co-O bond force constant (k) in the Co^3+^-O^2-^ structure at different temperatures.


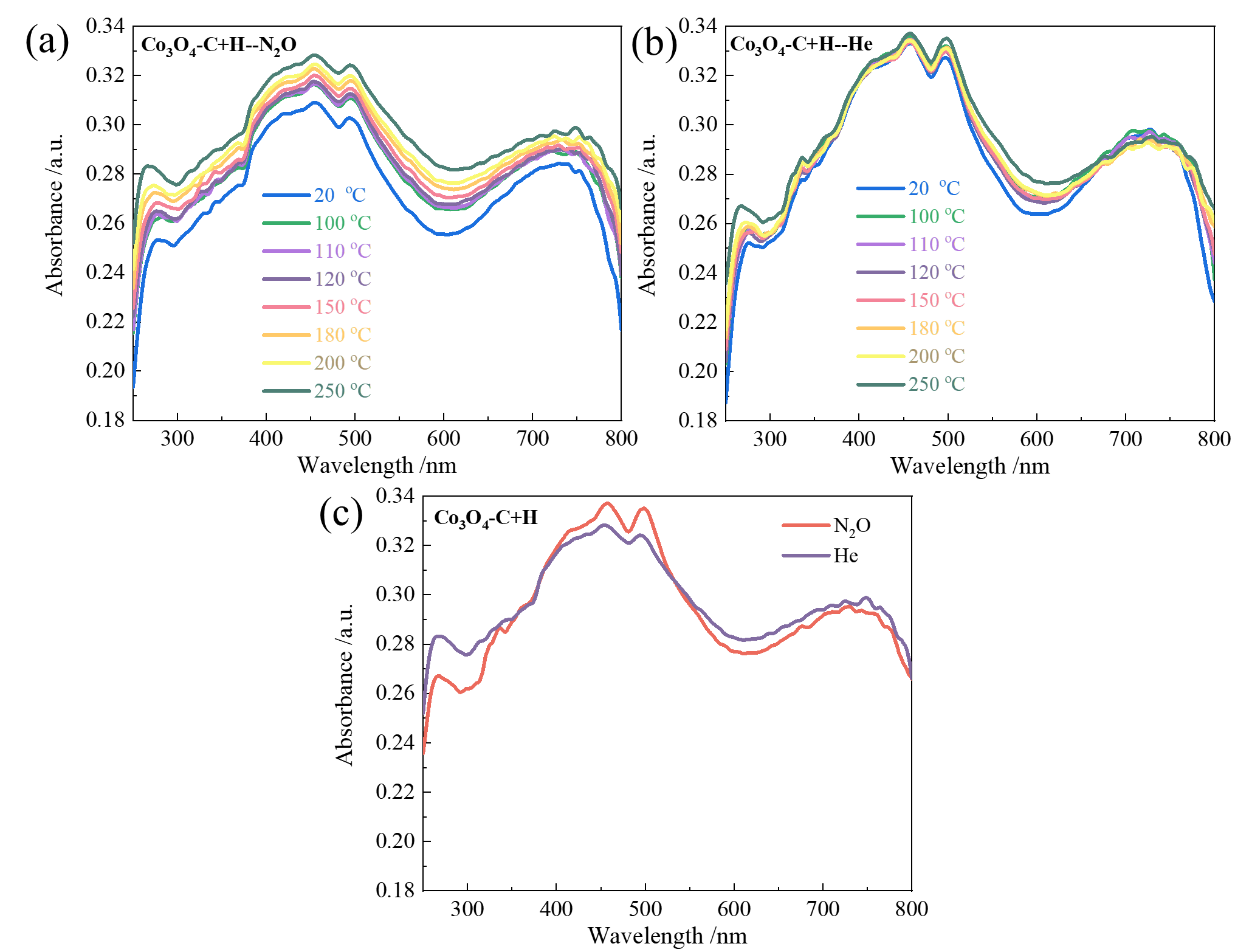


## Figure S9. In-situ UV-vis DRS for Co_3_O_4_-C+H catalyst under different conditions. a) 7 vol.% N_2_O/He atmosphere. b) Pure He atmosphere. c) Curves under two atmospheres at 250^o^C.


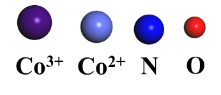


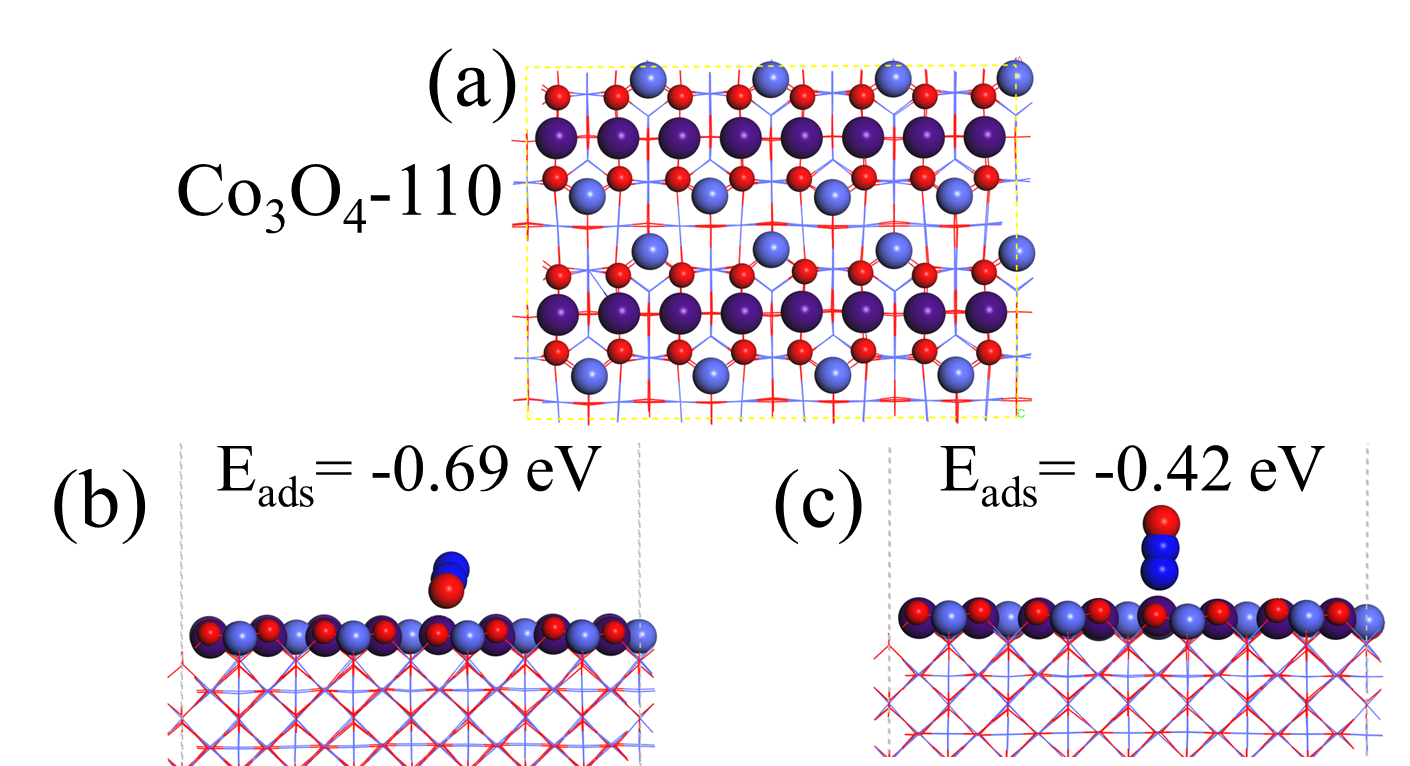


## Figure S10. The adsorption energies of N_2_O on Co^3+^ active sites at (110) facet of Co_3_O_4._ a) The top view of the 110 crystal facet of Co_3_O_4_. b) The O-terminal of N_2_O is adsorbed at the Co^3+^ site. c) The N-terminal of N_2_O is adsorbed at the Co^3+^ site.


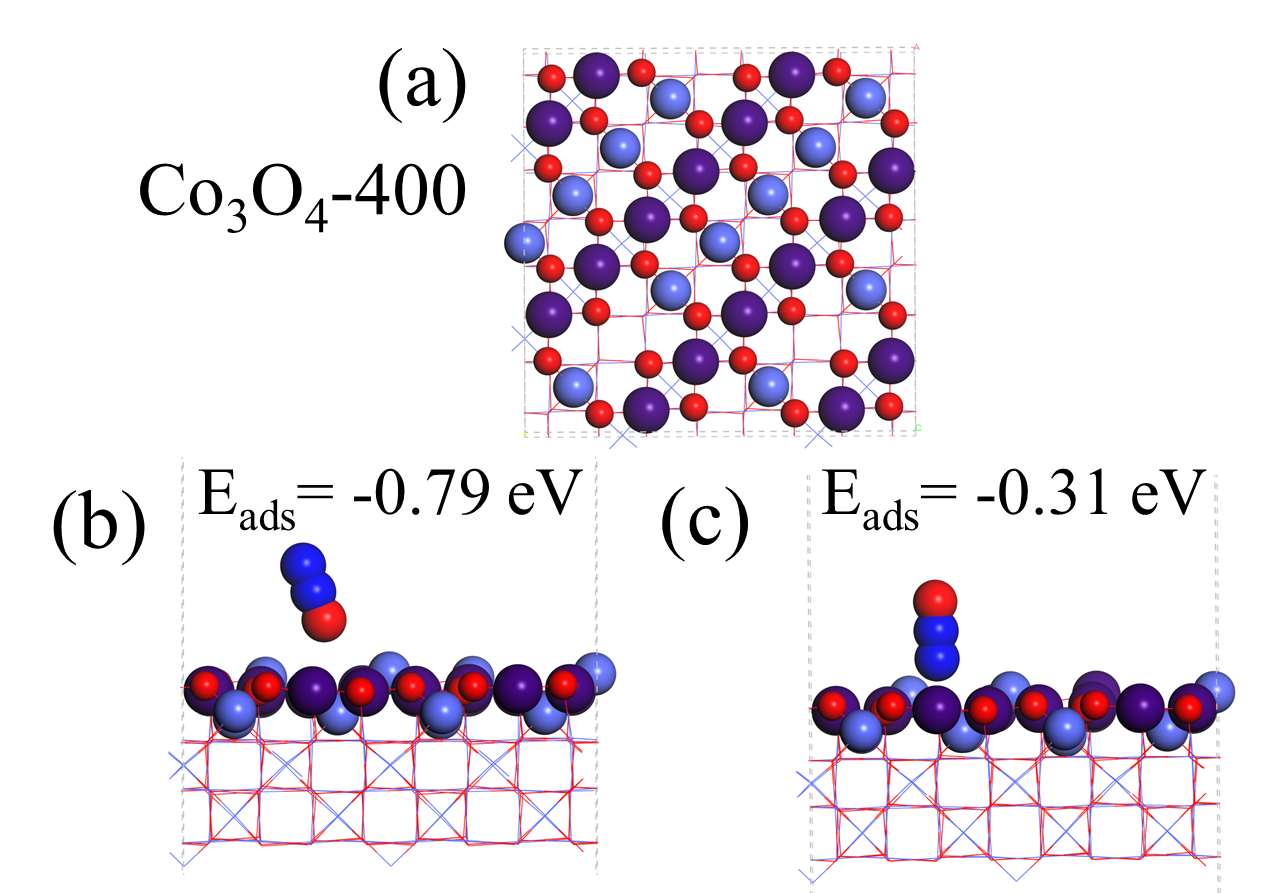


## Figure S11. The adsorption energies of N_2_O on Co^3+^ active sites at (400) facet of Co_3_O_4._ a) The top view of the (400) crystal facet of Co_3_O_4_. b) The O-terminal of N_2_O is adsorbed at the Co^3+^ site. c) The N-terminal of N_2_O is adsorbed at the Co^3+^ site.


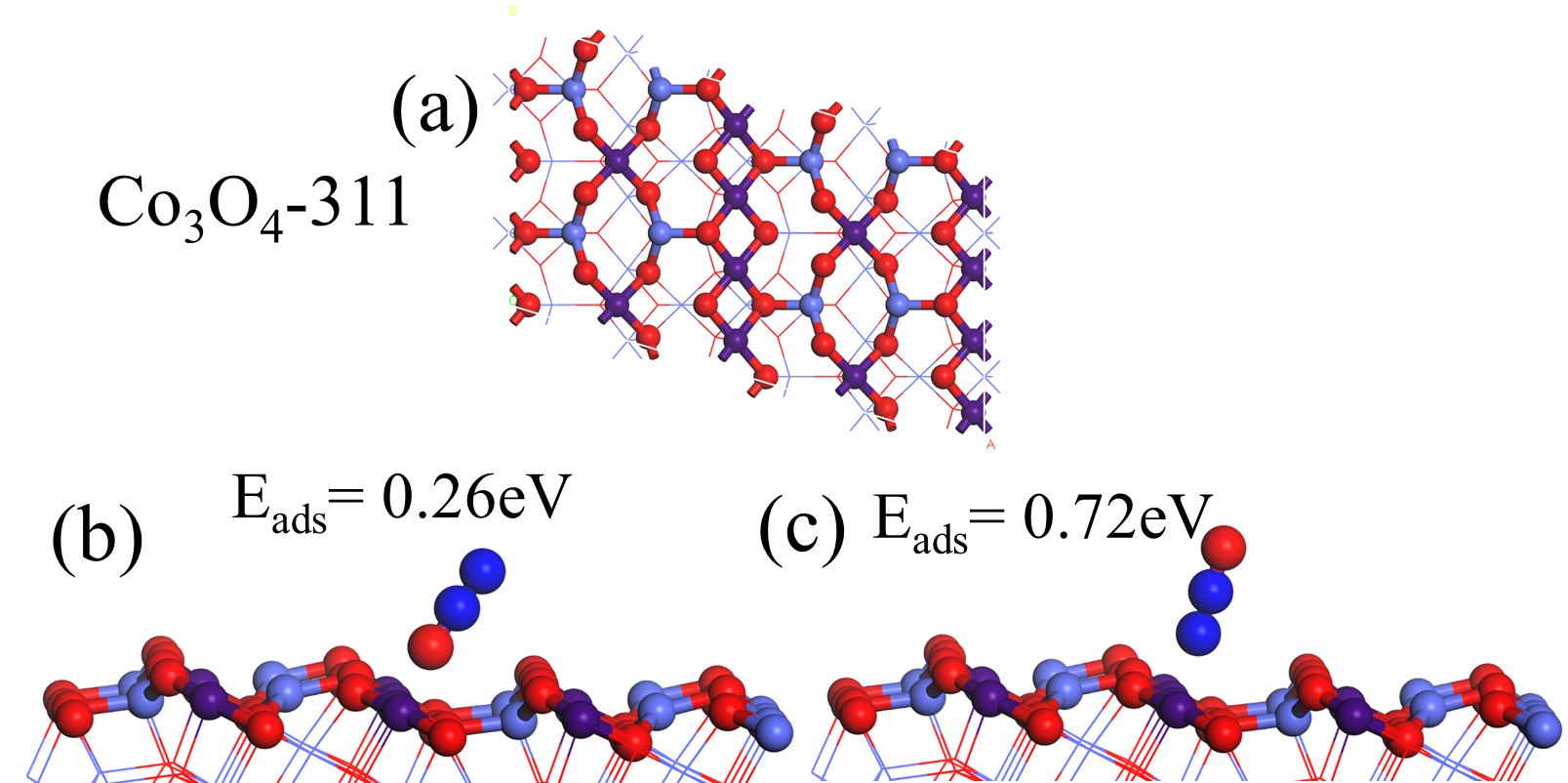


## Figure S12. The adsorption energies of N_2_O on Co^3+^ active sites at (311) facet of Co_3_O_4._ a) The top view of the (311) crystal facet of Co_3_O_4_. b) The O-terminal of N_2_O is adsorbed at the Co^3+^ site. c) The N-terminal of N_2_O is adsorbed at the Co^3+^ site.


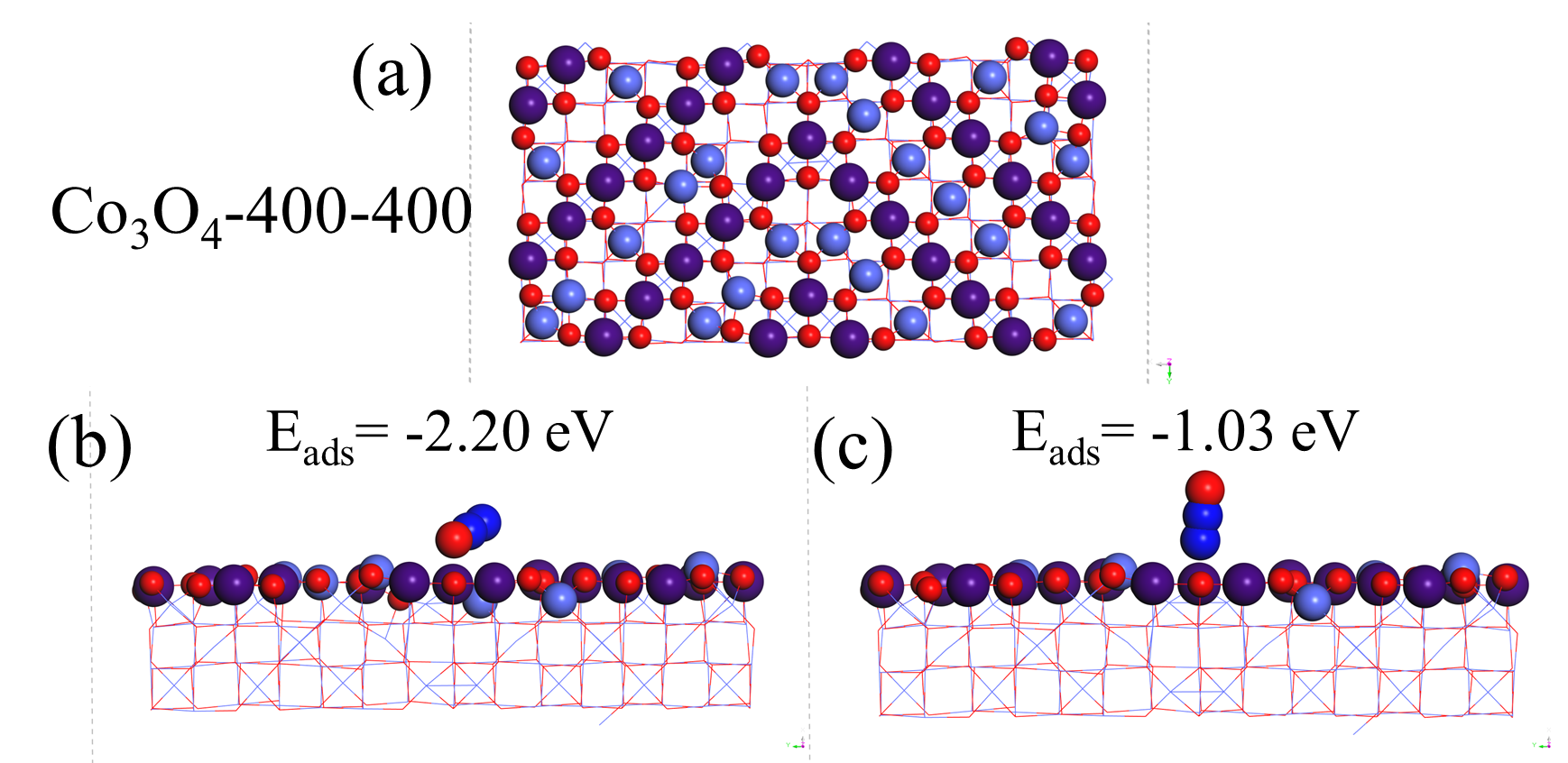


## Figure S13. The adsorption energies of N_2_O on Co^3+^ active sites at (400-400) facet of Co_3_O_4._ a) The top view of the 400-400 composite crystal facet of Co_3_O_4_. b) The O-terminal of N_2_O is adsorbed at the Co^3+^ site on the interface. c) The N-terminal of N_2_O is adsorbed at the Co^3+^ site on the interface.

## Figure S14. Effect of different atmosphere pretreatment on the performance of Co_3_O_4_-C+H catalyst.


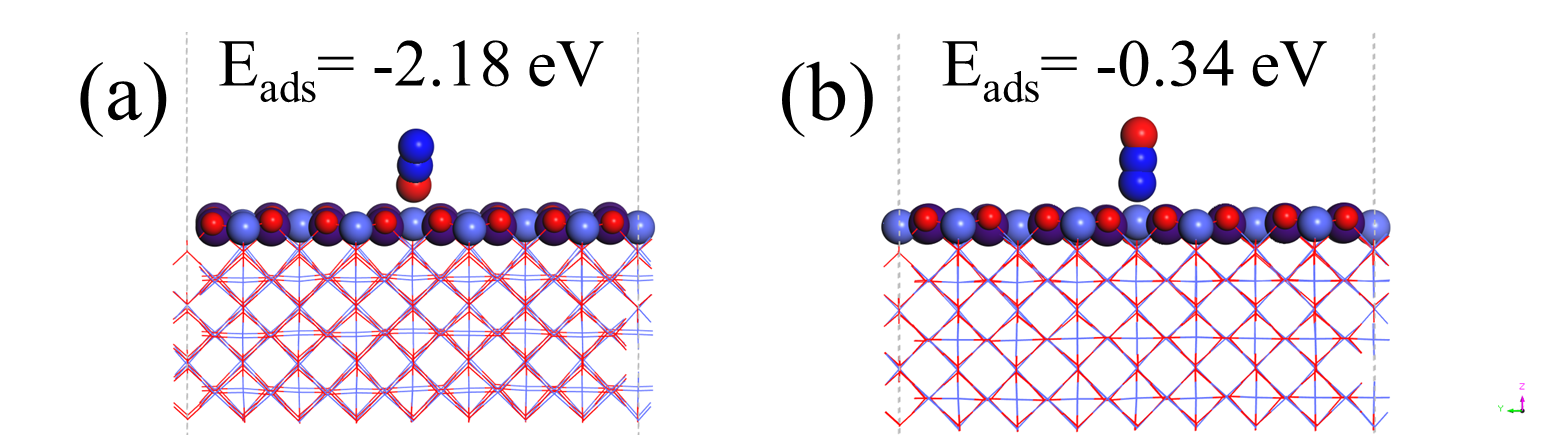


## Figure S15. The adsorption energies of N_2_O on Co^2+^ active sites at (110) facet of Co_3_O_4._ a) The O-terminal of N_2_O is adsorbed at the Co^2+^ site. b) The N-terminal of N_2_O is adsorbed at the Co^2+^ site.


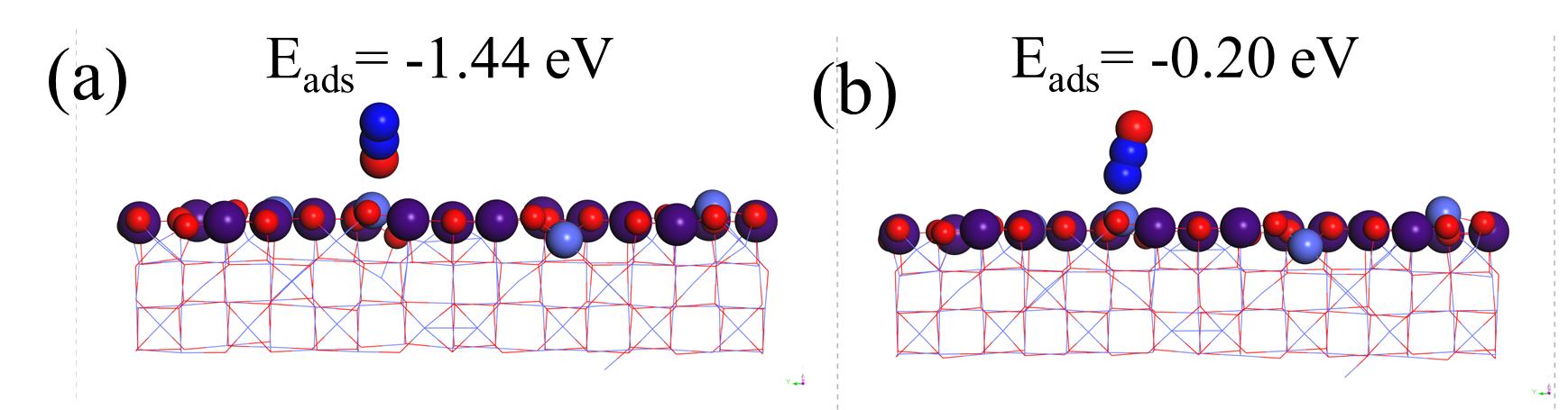


## Figure S16. The adsorption energies of N_2_O on Co^2+^ active sites at (400-400) facet of Co_3_O_4._ a) The O-terminal of N_2_O is adsorbed at the Co^2+^ site. b) The N-terminal of N_2_O is adsorbed at the Co^2+^ site.


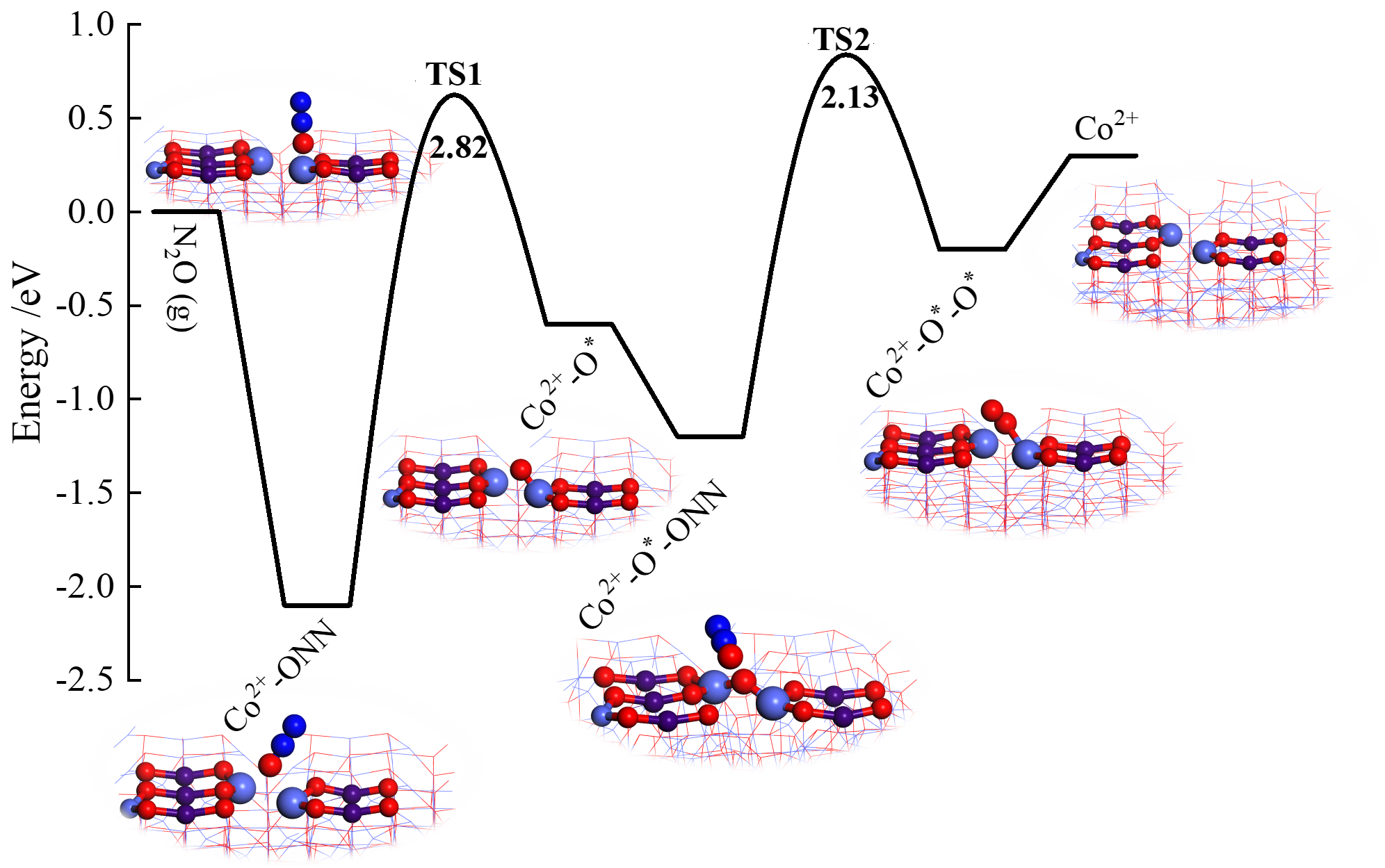


## Figure S17. The reaction energy diagrams of different N_2_O decomposition on the (110) facet.

## Figure S18. a) 400-400 structure containing O_v_. b) Adsorption energy of the O end of N_2_O on Co^3+^ sites containing O_v_. c) Reaction path of N_2_O on Co^3+^ sites containing O_v_. d) Adsorption energy of the O end of N_2_O on O_v_. e) Reaction path of N_2_O at O_v_.


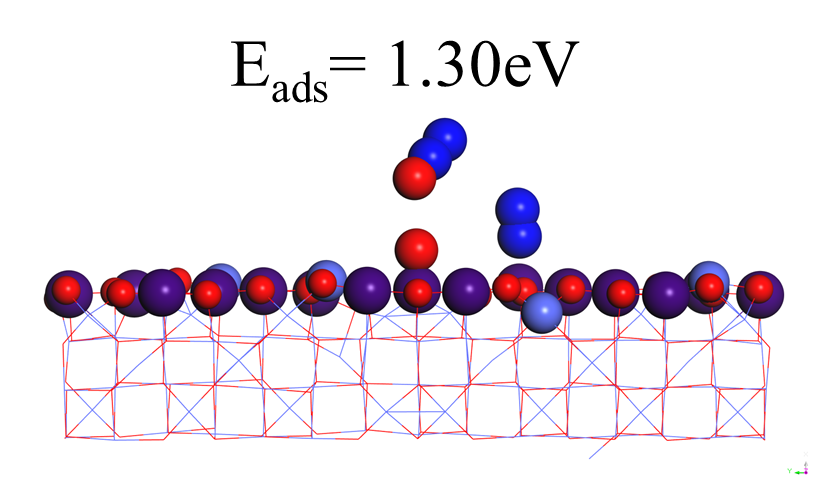


## Figure S19. The adsorption energies of the second N_2_O on Co^3+-^O* active sites at (400-400) facet of Co_3_O_4._


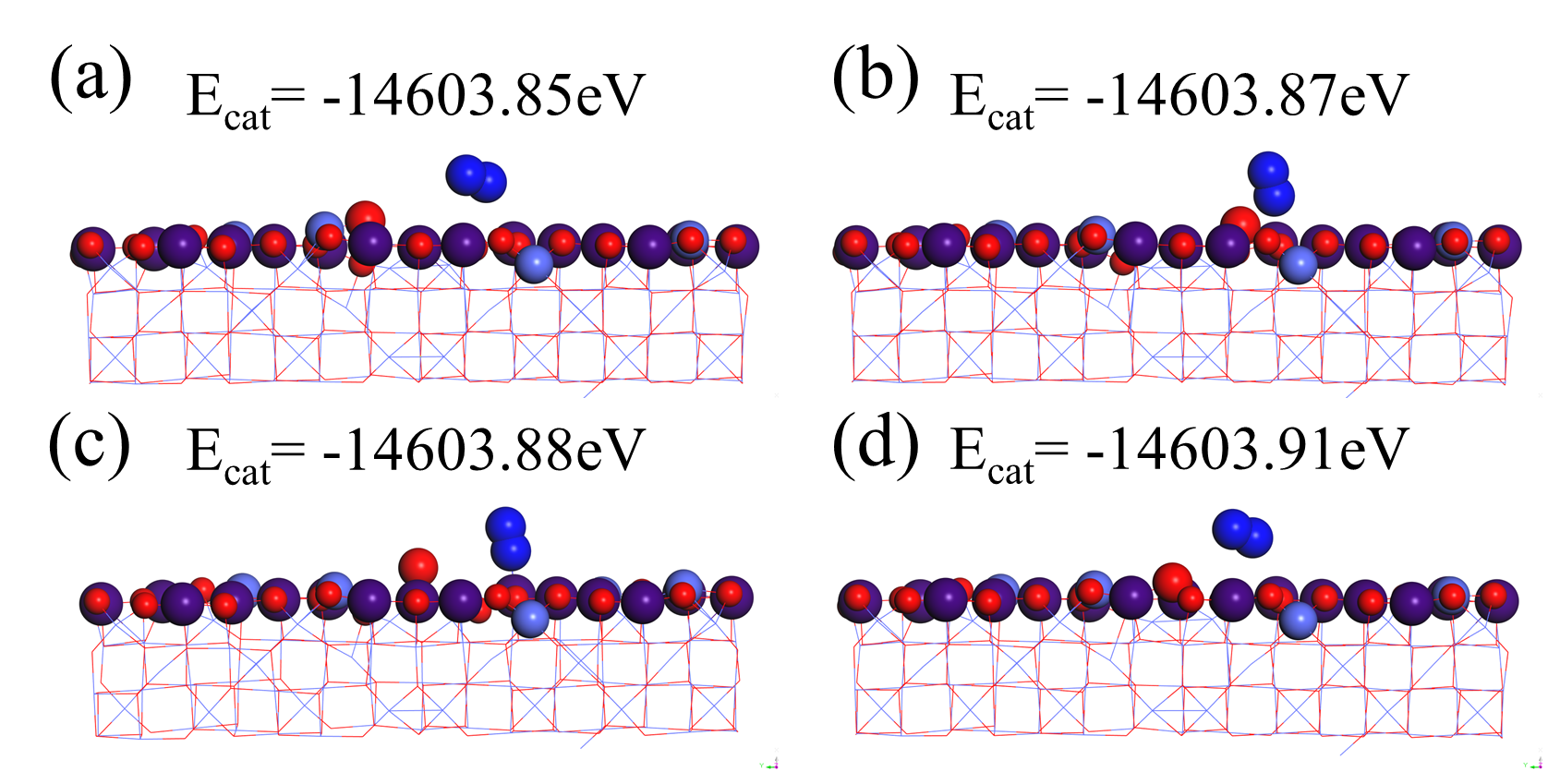


## Figure S20. The decomposed O* of the first N_2_O is transferred to the four adjacent oxygens next to Co^3+^. a) Left. b) Right. c) Front. d) Rear.


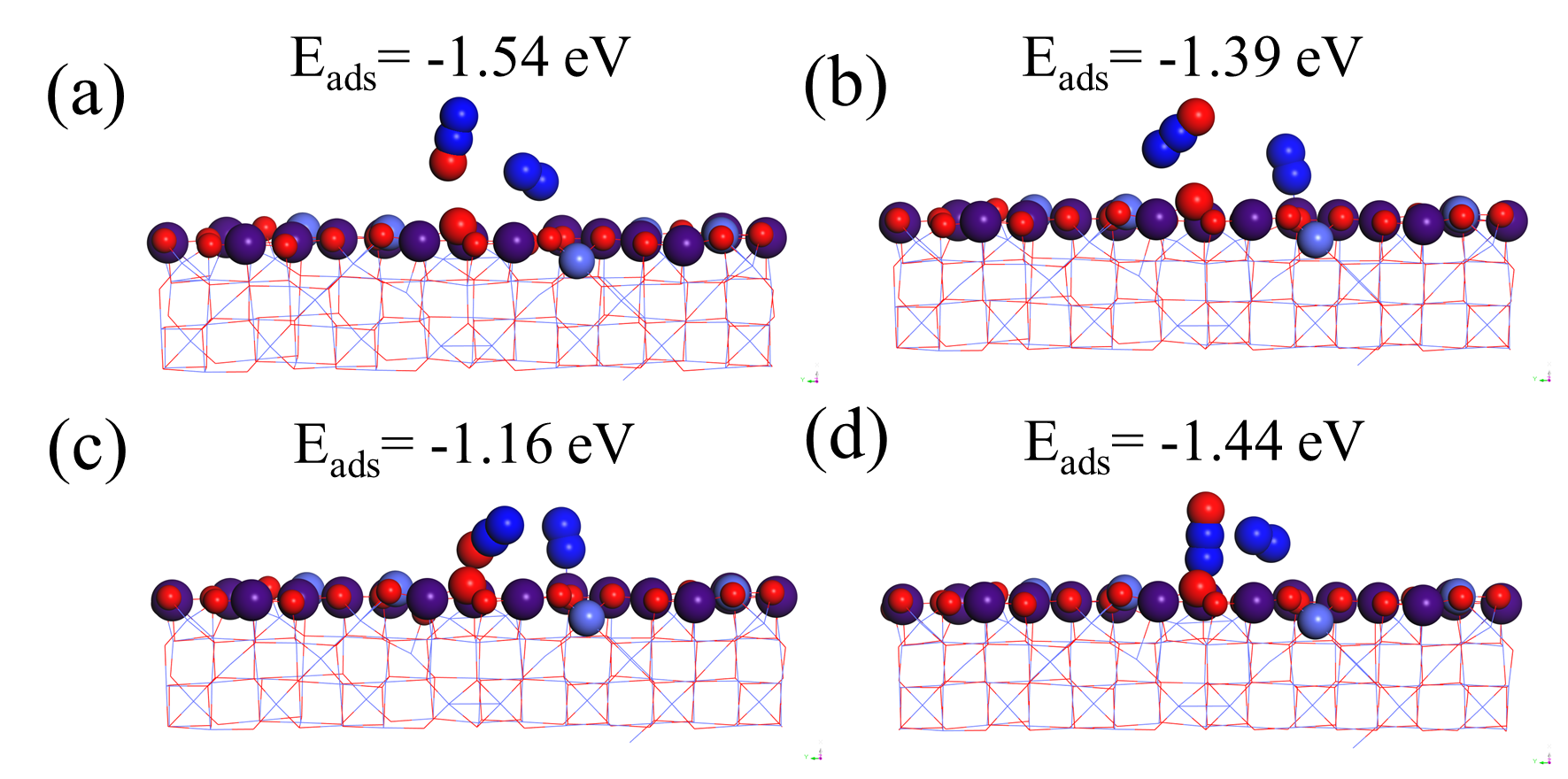


## Figure S21. The adsorption energies of the second N_2_O on new Co^3+-^O* active sites at (400-400) facet of Co_3_O_4._ a) The O-terminal of the second N_2_O is adsorbed at the O* site on the interface. b) The N-terminal of the second N_2_O is adsorbed at the O* site on the interface. c) The O-terminal of the second N_2_O is adsorbed at the Co^3+^ site on the interface. d) The N-terminal of the second N_2_O is adsorbed at the Co^3+^ site on the interface.


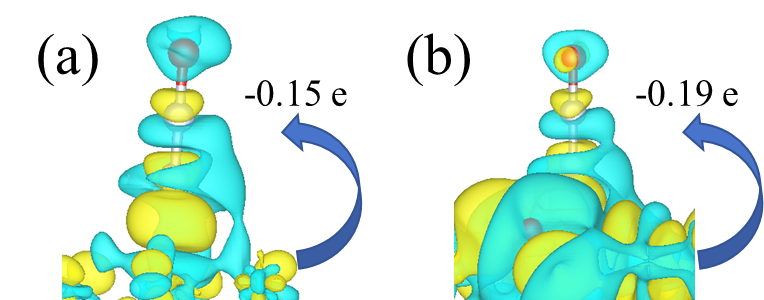


## Figure S22. The charge density difference (CDD) of N_2_O adsorption on different structures of Co_3_O_4_(400-400), the cyan area represents electron deficiency and the yellow area represents electron enrichment. a) The N-terminal of the first N_2_O is adsorbed at the Co^3+^ site on the interface. b) The N-terminal of the second N_2_O is adsorbed at the Co^3+^ site on the new active Co^3+^-O* motif.

## Figure S23. The XPS spectra of O 1s of Co_3_O_4_-C+H catalyst after reaction.

# Supplementary Tables

## Table S1. The T_90_ of different catalysts reported in the literatures.

| **Catalysts** | **Reaction Conditions** | **T_90_ /^o^C** | **Ref** |
| --- | --- | --- | --- |
| Ir_0.5_/CeO_2_-Al_2_O_3_ | 1000 ppm N_2_O/He, GHSV = 40000 h^−1^ | 525 | 17 |
| Pd_2.6_/γAl_2_O_3_ | 1% N_2_O/He, GHSV = 76690 h^−1^ | 600 | 18 |
| Pd_1.0_/LaCoO_3_ | 1000 ppm N_2_O/He, GHSV = 10000 h^−1^ | 500 | 19 |
| Rh/CeO_2_ | 200 ppm N_2_O/N_2_, GHSV = 30000 h^−1^ | 400 | 23 |
| Co_3_O_4_ | 1000 ppm N_2_O/Ar, GHSV = 30000 h^−1^ | 520 | 21 |
| Pr_0.06_-Co_3_O_4_ | 2000 ppm N_2_O/Ar, GHSV = 60000 h^−1^ | 367 | 22 |
| Co_3_O_4_/CeO_2_ | 1000 ppm N_2_O/Ar, GHSV = 40000 h^−1^ | 450 | 24 |
| CuO-Co_3_O_4_ | 1000 ppm N_2_O/N_2_, GHSV = 60000 h^−1^ | 350 | 25 |
| K-Co_2.6_Zn_0.4_O_4_\|α-Al_2_O_3_ | 5% N_2_O/He, GHSV = 7000 h^−1^ | 500 | 26 |
| Co_3_O_4_ on open-cell metallic foam | 1000 ppm N_2_O/N_2_, GHSV = 6800 h^−1^ | 525 | 27 |
| Co_3_O_4_/g-CN | 1000 ppm N_2_O/Ar, GHSV = 1000 h^−1^ | 360 | 28 |
| Co_3_O_4_\|α-Al_2_O_3_ | 5% N_2_O/He, GHSV = 1600 h^−1^ | 500 | 29 |
| Fe-ZSM-5 | 1500 ppm N_2_O/Ar, GHSV = 35000 h^−1^ | 620 | 30 |
| Cu-ZSM-5 | 1% N_2_O/He, GHSV = 6000 h^−1^ | 580 | 31 |
| Co-ZSM-5 | 30% N_2_O/He, GHSV = 30000 h^−1^ | 500 | 32 |

## Table S2. BET for Co_3_O_4_-C, Co_3_O_4_-H, Co_3_O_4_-C+H catalysts.

| **Catalysts** | **Surface Area**  **（m^2^/g）** | **Pore Volume**  **（cm^3^/g）** | **Pore Size**  **（nm）** |
| --- | --- | --- | --- |
| Co_3_O_4_-C | 16.39 | 0.05 | 4.54 |
| Co_3_O_4_-H | 19.71 | 0.14 | 16.01 |
| Co_3_O_4_-C+H  (pH=9,60 mL/h) | 13.75 | 0.08 | 13.99 |

## Table S3. Kinetic parameters of N_2_O catalytic decomposition over Co_3_O_4_ catalysts.

| **Catalysts** | **T**  **（K）** | **r**  **（mol.s^-1^.g^-1^）** | **TOF**  **（s^-1^）** | **E_a_**  **（kJ.mol^-1^）** |
| --- | --- | --- | --- | --- |
| Co_3_O_4_-C | 623 | 4.20x10^-6^ | 1.12x10^-4^ | 78.96 |
|  | 573 | 1.41x10^-6^ | 3.75x10^-4^ |  |
|  | 523 | 2.52x10^-7^ | 6.72x10^-5^ |  |
| Co_3_O_4_-H | 623 | 4.30x10^-6^ | 9.89x10^-4^ | 73.18 |
|  | 573 | 2.29x10^-6^ | 5.07x10^-4^ |  |
|  | 523 | 1.19x10^-6^ | 2.74x10^-4^ |  |
| Co_3_O_4_-C+H  (pH=9.5,60+120 mL/h) | 573 | 5.53x10^-5^ | 1.89x10^-2^ | 44.26 |
|  | 523 | 2.43x10^-5^ | 8.30x10^-3^ |  |
|  | 473 | 7.95x10^-6^ | 2.27x10^-3^ |  |

# References

1. F. Lin, T. Andana, Y. Wu, J. Szanyi, Y. Wang, F. Gao, *J. Catal*. **2021**, 401, 7.
2. S. Li, J. Zhao, Z. Song, H. Wang, T. Zhang, J. Liu, Q. Jiang, *Fuel*. **2024**, 362, 130745.
3. F. Gao, E. D. Walter, M. Kollar, Y. Wang, J. Szanyi, C. H. F. Peden, *J. Catal.* **2014**, 319, 1.
4. C.-Y. Li, J.-B. Le, Y.-H. Wang, S. Chen, Z.-L. Yang, J.-F. Li, J. Cheng, Z.-Q. Tian, *Nat. Mater*. **2019**, 18, 697.
5. J. VandeVondele, M. Krack, F. Mohamed, M. Parrinello, T. Chassaing, J. Hutter, *Comput. Phys. Commun*. **2005**, 167, 103.
6. J. Hutter, M. Iannuzzi, F. Schiffmann, J. VandeVondele, *WIREs Comput. Mol. Sci*. **2014**, 4, 15.
7. Y. Peng, H. Hajiyani, R. Pentcheva, *ACS Catal*. **2021**, 11, 5601.
8. Y. Long, X. Zhu, C. Gao, W. Si, J. Li, Y. Peng, *Nat. Commun*. **2025**, 16, 1048.
9. S. Goedecker, M. Teter, J. Hutter, *Phys. Rev. B*. **1996**, 54, 1703.
10. Y. Liu, Y. Peng, M. Naschitzki, S. Gewinner, W. Schöllkopf, H. Kuhlenbeck, R. Pentcheva, B. Roldan Cuenya, *Angew. Chem. Int. Ed*. **2021**, 60, 16514.
11. C. Hartwigsen, S. Goedecker, J. Hutter, *Phys. Rev. B*. **1998**, 58, 3641.
12. J. VandeVondele, J. Hutter, *J. Chem. Phys*. **2007**, 127, 114105.
13. J. P. Perdew, K. Burke, M. Ernzerhof, *Phys. Rev. Lett*. **1996**, 77, 3865.
14. D. Sheppard, P. Xiao, W. Chemelewski, D. D. Johnson, G. Henkelman, *Chem. Phys*. **2012**, 136, 074103.
15. G. Henkelman, B. P. Uberuaga, H. Jónsson, *Chem.Phys*. **2000**, 113, 9901.
16. L. Xiao, S. Mou, W. Dai, W. Yang, Q. Cheng, S. Liu, F. Dong, *Angew. Chem. Int. Ed*. **2024**, 63, e202319135.
17. E. Pachatouridou, E. Papista, A. Delimitis, M. A. Vasiliades, A. M. Efstathiou, M. D. Amiridis, O. S. Alexeev, D. Bloom, G. E. Marnellos, M. Konsolakis, E. Iliopoulou, *Appl. Catal. B: Environ*. **2016**, 187, 259.
18. N. Richards, J. H. Carter, E. Nowicka, L. A. Parker, S. Pattisson, Q. He, N. F. Dummer, S. Golunski, G. J. Hutchings, *Appl. Catal. B: Environ*. **2020**, 264, 118501.
19. J. P. Dacquin, C. Dujardin, P. Granger, *J. Catal*. **2008**, 253, 37.
20. K. Yuzaki, T. Yarimizu, K. Aoyagi, S.-i. Ito, K. Kunimori, *Catal. Today*. **1998**, 45, 129.
21. X. Hu, Y. Wang, R. Wu, Y. Zhao, *Mol. Catal*. **2021**, 509, 111656.
22. H. Liu, S. Yang, G. Wang, H. Liu, Y. Peng, C. Sun, J. Li, J. Chen, *Environ. Sci. Technol*. **2022**, 56, 16325.
23. Y. Li, A. Sundermann, O. Gerlach, K.-B. Low, C. Zhang, X. Zheng, H. Zhu, S. Axnanda, *Catal. Today*. **2020**, 355, 608.
24. M. Lykaki, E. Papista, N. Kaklidis, S. A. C. Carabineiro, M. Konsolakis, *Catalysts*. **2019**, 9, 233.
25. S. Xiong, J. Chen, N. Huang, S. Yang, Y. Peng, J. Li, *Environ. Sci. Technol*. **2019**, 53, 10379.
26. G. Grybek, S. Wojcik, P. Legutko, J. Grybos, P. Indyka, B. Leszczynski, A. Kotarba, Z. Sojka, *Appl. Catal. B*. **2017**, 205, 597.
27. P. H. Ho, K. S. Da Costa, G. S. de Luna, M. Jablonska, F. Ospitali, F. Di Renzo, G. Delahay, G. Fornasari, A. Vaccari, R. Palkovits, P. Benito, *Chem. Eng. Res*. **2022**, 188, 166.
28. X. Hu, Y. Wang, R. Wu, Y. Zhao, *Appl. Surf. Sci*. **2021**, 538, 148157.
29. S. Wojcik, G. Ercolino, M. Gajewska, C. W. M. Quintero, S. Specchia, A. Kotarba, *Chem. Eng. J*. **2019**, 377, 120088.
30. G. He, B. Zhang, H. He, X. Chen, Y. Shan, Sci. Total Environ. **2019**, 673, 266-271.
31. S. A. Yashnik, A. V. Salnikov, N. T. Vasenin, V. F. Anufrienko, Z. R. Ismagilov, *Catal. Today*. **2012**, 197, 214.
32. B. Kang, M. Li, Z. Di, X. Guo, Y. Wei, J. Jia, R. Zhang, *Catal. Today***.** ***2022****, 402*, 17.
